# Biphasic zinc compartmentalisation in a human fungal pathogen

**DOI:** 10.1371/journal.ppat.1007013

**Published:** 2018-05-04

**Authors:** Aaron C. Crawford, Laura E. Lehtovirta-Morley, Omran Alamir, Maria J. Niemiec, Bader Alawfi, Mohammad Alsarraf, Volha Skrahina, Anna C. B. P. Costa, Andrew Anderson, Sujan Yellagunda, Elizabeth R. Ballou, Bernhard Hube, Constantin F. Urban, Duncan Wilson

**Affiliations:** 1 Medical Research Council Centre for Medical Mycology at the University of Aberdeen, Aberdeen Fungal Group, Institute of Medical Sciences, Foresterhill, Aberdeen, United Kingdom; 2 School of Biological Sciences, University of East Anglia, Norwich, United Kingdom; 3 Department of Clinical Microbiology, Umeå Centre for Microbial Research and Molecular Infection Medicine Sweden, Umeå University, Umeå, Sweden; 4 Research Group Microbial Immunology, Leibniz Institute for Natural Product Research and Infection Biology–Hans Knoell Institute, Jena, Germany; 5 Center for Sepsis Control and Care (CSCC), University Hospital, Jena, Germany; 6 Department of Microbial Pathogenicity Mechanisms, Leibniz Institute for Natural Product Research and Infection Biology–Hans Knoell Institute, Jena, Germany; 7 Institute of Microbiology and Infection, and School of Biosciences, University of Birmingham, Birmingham, United Kingdom; 8 Friedrich Schiller University, Jena, Germany; Geisel School of Medicine at Dartmouth, UNITED STATES

## Abstract

Nutritional immunity describes the host-driven manipulation of essential micronutrients, including iron, zinc and manganese. To withstand nutritional immunity and proliferate within their hosts, pathogenic microbes must express efficient micronutrient uptake and homeostatic systems. Here we have elucidated the pathway of cellular zinc assimilation in the major human fungal pathogen *Candida albicans*. Bioinformatics analysis identified nine putative zinc transporters: four cytoplasmic-import Zip proteins (Zrt1, Zrt2, Zrt3 and orf19.5428) and five cytoplasmic-export ZnT proteins (orf19.1536/Zrc1, orf19.3874, orf19.3769, orf19.3132 and orf19.52). Only Zrt1 and Zrt2 are predicted to localise to the plasma membrane and here we demonstrate that Zrt2 is essential for *C*. *albicans* zinc uptake and growth at acidic pH. In contrast, *ZRT1* expression was found to be highly pH-dependent and could support growth of the *ZRT2*-null strain at pH 7 and above. This regulatory paradigm is analogous to the distantly related pathogenic mould, *Aspergillus fumigatus*, suggesting that pH-adaptation of zinc transport may be conserved in fungi and we propose that environmental pH has shaped the evolution of zinc import systems in fungi. Deletion of *C*. *albicans ZRT2* reduced kidney fungal burden in wild type, but not in mice lacking the zinc-chelating antimicrobial protein calprotectin. Inhibition of *zrt2Δ* growth by neutrophil extracellular traps was calprotectin-dependent. This suggests that, within the kidney, *C*. *albicans* growth is determined by pathogen-Zrt2 and host-calprotectin. As well as serving as an essential micronutrient, zinc can also be highly toxic and we show that *C*. *albicans* deals with this potential threat by rapidly compartmentalising zinc within vesicular stores called zincosomes. In order to understand mechanistically how this process occurs, we created deletion mutants of all five ZnT-type transporters in *C*. *albicans*. Here we show that, unlike in *Saccharomyces cerevisiae*, *C*. *albicans* Zrc1 mediates zinc tolerance via zincosomal zinc compartmentalisation. This novel transporter was also essential for virulence and liver colonisation *in vivo*. In summary, we show that zinc homeostasis in a major human fungal pathogen is a multi-stage process initiated by Zrt1/Zrt2-cellular import, followed by Zrc1-dependent intracellular compartmentalisation.

## Author summary

All living organisms must secure certain trace metals such as iron and zinc in their diets. For the microbes that infect us, the source of these micronutrients is the tissues of their host. However, mammals have developed sophisticated mechanisms to manipulate microbial access to trace metals–a process called *nutritional immunity*. Therefore, successful pathogenic microorganisms must have evolved mechanisms to counteract nutritional immunity and acquire micronutrients in order to grow within their hosts and cause disease. This struggle for micronutrients represents a key host-pathogen battleground. In this study we demonstrate how the major human fungal pathogen, *Candida albicans*, acquires and stores zinc from its environment. We find that the mechanistic basis of zinc uptake is highly dependent on the acidity of the surrounding environment. Interestingly, this pH-dependence appears conserved in the fungal kingdom and we propose a potential framework for the evolution of zinc uptake in extant fungal species. Moreover, following cellular assimilation, *C*. *albicans* shuttles this potentially toxic transition metal into subcellular compartments called zincosomes. We also show that both zinc uptake and compartmentalisation are critical for *C*. *albicans* growth, both under laboratory conditions and in experimental models of invasive candidiasis.

## Introduction

Certain trace metals such as iron and zinc (collectively termed micronutrients) are essential for cellular life, and at least a third of all proteins interact with a metal cofactor [1]. Zinc is particularly important for eukaryotes as around 9% of their proteomes require this metal for function [2]. However, these essential metals can also be highly toxic to cells, and precise metal ion homeostasis is critical for survival. Pathogenic microorganisms face a complicated relationship with micronutrients as the mammalian host uses both high antimicrobial concentrations of metals, as well as metal sequestration to kill microbes or inhibit their growth. Collectively, these processes are known as *nutritional immunity* (4). The “battle for iron” is an established paradigm in host-pathogen interactions [3] and, more recently, important roles for manganese, copper and zinc have emerged within the framework of nutritional immunity [4]. Zinc in particular represents a double-edged sword for potentially invasive species. Botella *et al*. established that phagocytosed *Mycobacterium tuberculosis* cells experience acute zinc toxicity within macrophages, and that intracellular survival is reliant on heavy metal efflux P-type-ATPase activity [5]. In other host niches, zinc availability is extremely limited due to systemic zincaemia or locally produced zinc-chelating agents such as calprotectin. In these environments, efficient zinc uptake is crucial for pathogenicity, and a number of recent studies have demonstrated the importance of the *znuABC* high affinity zinc importer for bacterial virulence [4,6].

Fungi do not appear to encode ABC transport systems for zinc acquisition. Instead, eukaryotic zinc transport can be mediated by members of two protein families: the Zip and ZnT transporters, which transport zinc into and out of the cytoplasm, respectively [7]. In the model yeast *S*. *cerevisiae*, Zip family members have been shown to assimilate zinc from the environment or to export zinc from intracellular organelles such as the vacuole [8–10]. In contrast, ZnT proteins play roles in organellar zinc accumulation. In *S*. *cerevisiae*, the major target for excess zinc is the vacuole [11], as well as small vesicular zinc storage compartments called zincosomes [12]. Whilst vacuolar zinc import is mediated by the ZnT-type transporter Zrc1 [13], the mechanism of fungal intracellular zincosomal zinc compartmentalisation is not known [12].

Predicted plasma membrane Zip transporters have now been characterised in the major human fungal pathogens *Aspergillus fumigatus*, *H*. *capsulatum*, *Cryptococcus* *neoformans* and *C. gattii* [14–17], and Zip transporter mutants for all four species exhibit attenuated virulence, underscoring the importance of zinc uptake for fungal, as well as bacterial pathogenicity.

*Candida albicans* is a normal commensal member of the human gastrointestinal microbiota and other mucosal surfaces, a common cause of mucosal infections, and a serious invasive pathogen in certain patient groups [18]. In fact, invasive candidiasis, predominantly caused by *C*. *albicans*, affects more than a quarter of a million individuals each year and is responsible for at least 50,000 deaths annually [19]. We have previously shown that this fungus can scavenge zinc via the secreted protein Pra1 and that this “zincophore” system is important for host cell damage in tissue culture infection models [20]. However, a *pra1Δ* mutant is hyper-virulent in a mouse model of infection as it also serves as a ligand for neutrophil alphaMbeta2 [21,22].

In this study we have functionally dissected zinc transport in *C*. *albicans*. We identified nine putative zinc transporters including two predicted plasma membrane Zip proteins, Zrt1 and Zrt2, as well as five ZnT proteins. Regulatory and functional analysis demonstrates that pH-dependent adaptation to zinc limitation may be conserved in fungi, but that distinct transporter subclasses differentially contribute to growth *in vivo* for different human pathogenic species. Moreover, for the first time, we define a molecular mechanism of zincosomal zinc accumulation in a human fungal pathogen.

## Results and discussion

### Identification of zinc importers in *Candida albicans*

Zinc transport in eukaryotes can be mediated by members of the Zip and ZnT protein families, which transport their substrate to or from the cytoplasm, respectively [7]. In order to determine how *C*. *albicans* acquires zinc from its environment, we first focussed on Zip transporters. Using the FungiDB [23] InterPro domain-finder (PFAM: PF02535; http://fungidb.org/fungidb/) we identified four Zip-type *C*. *albicans* proteins. Only Zrt1 and Zrt2 are predicted plasma membrane transporters. In contrast, Zrt3 and orf19.5428 share similarity with *S*. *cerevisiae* Zrt3 (vacuolar zinc) and Atx2 (Golgi manganese) transporters. We had previously generated a *C*. *albicans zrt1Δ* mutant as part of our efforts to characterise the fungal zincophore, Pra1 [20]. In this previous study we found that Zrt1 was essential for the reassociation of soluble Pra1 to the fungal cell surface, indicating that Zrt1 is likely cell surface-localised. *ZRT2*, on the other hand, was (at time of writing) annotated in the *Candida* Genome Database (www.candidagenome.org/) [24] as a possibly essential gene (Aaron Mitchell, personal communication to the CGD). Indeed, our initial attempts to delete the second allele of *ZRT2* were unsuccessful (the first 104 second-round clones retained their second allele of *ZRT2*). Supplementation of the transformation selection medium with 1 mM ZnSO_4_ permitted the successful isolation of a *C*. *albicans zrt2Δ* homozygous mutant, suggesting that *ZRT2* is conditionally essential.

### Zrt1 and Zrt2—pH dependent zinc acquisition

**Fig 1:**
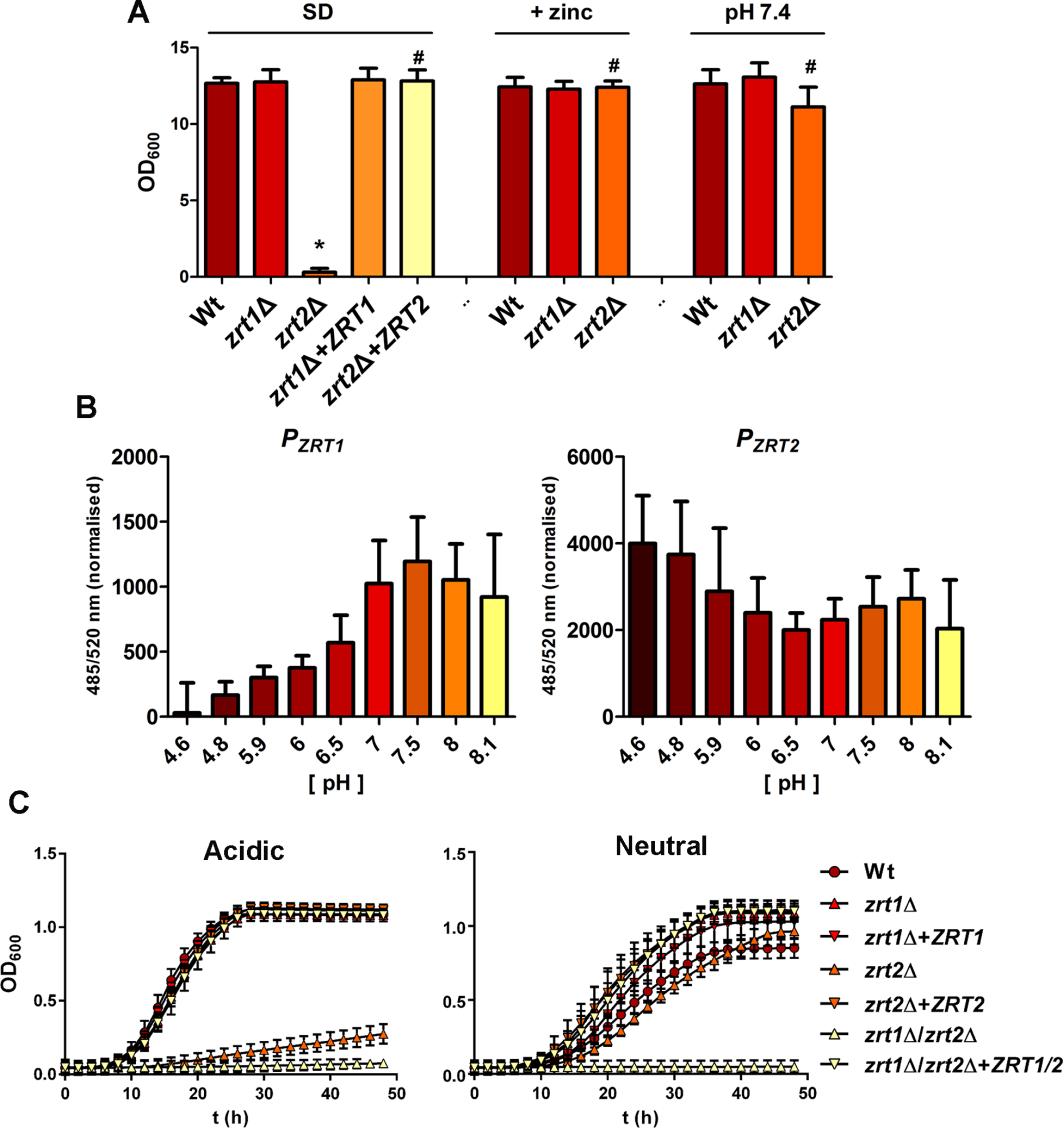
pH-dependent functionality and regulation of Zrt1 and Zrt2 in *C*. *albicans*. (**A**) Zrt2 is essential in acidic medium. Indicated strains, precultured in YPD, were washed and cultured in SD (YNB+glucose) medium alone, or supplemented with 100 μM ZnSO4 or with 50 mM HEPES pH 7.4. Asterisks indicate statistical significance compared to the wild type; # indicates statistical significance compare to the *zrt2*Δ in SD; P <0.05. (**B**) *ZRT1* promoter activity is pH regulated and *ZRT2* is constitutively expressed under zinc limitation. (*P*_*ZRT1*_-GFP and *P*_*ZRT2*_-GFP reporter strains in LZM buffered to indicated pH values). LZM was used due to lower green autofluorescence. Experiment performed three times. (**C**) Double deletion of *ZRT1* and *ZRT2* precludes growth at both acidic and neutral alkaline pH. Strains were cultured as in (A) and growth kinetics measured over 48 h in a microtitre plate. Experiment performed twice in triplicate.

Subsequent attempts to culture *C*. *albicans zrt2Δ* in SD (YNB + glucose) medium (the minimal yeast growth medium, routinely used for the selection of transformants) failed, indicating that *ZRT2* is indeed essential for growth under this laboratory condition. Consistent with conditional essentiality, growth of *zrt2Δ* was restored to wild type levels via zinc supplementation or by genetic complementation with a single copy of *ZRT2* (**Fig 1A**). Deletion of *ZRT1* did not impact growth in SD medium. We also tested growth in liquid and agar hyphae-inducing medium and under biofilm conditions, but observed no difference between wild type and *zrt1Δ* or *zrt2Δ* strains (**S1 Fig and S2 Fig**).

In the pathogenic mould *A*. *fumigatus*, Zrt1 and Zrt2 orthologues (ZrfC and ZrfB) are required for zinc uptake at neutral/alkaline and acidic pH, respectively [25–27]. Although pH-dependent zinc transport has not been reported in the more closely related yeast, *S*. *cerevisiae*, a previous study has indicated that *C*. *albicans ZRT1* and *ZRT2* are also pH-regulated [28]. As SD minimal medium has a native pH of **~**4.8, we tested the effect of neutralising the growth medium. Buffering the medium to pH 7.4 restored growth of *zrt2Δ*, and had no adverse effect on *zrt1Δ*, which again grew to wild type levels (**Fig 1A**). Similar pH dependent growth patterns and zinc rescue effects were observed in synthetic limited zinc medium (**S3 Fig**).

As *C*. *albicans* encodes only two predicted plasma membrane zinc importers, these data indicated that, in laboratory medium, Zrt1 can support growth at neutral-alkaline pH, whilst Zrt2 is essential for growth at acidic pH. Based on these growth patterns, we hypothesised that *ZRT1* is specifically expressed at neutral/alkaline pH, whilst *ZRT2* expression is pH-independent. We note that this regulatory and functional model aligns more closely with that of the pathogenic mould *A*. *fumigatus* [25–27].

To test this hypothesis we constructed *C*. *albicans* reporter strains with GFP [29] expression driven from either the *ZRT1* or *ZRT2* promoters. **Fig 1B** shows the expression profiles of *P*_*ZRT1*_ and *P*_*ZRT2*_ in low-zinc medium at a range of environmental pH values. GFP fluorescence driven by *P*_*ZRT1*_ activity was low at pH 4.6. However, as the media was neutralised, fluorescence increased. Expression was 40-fold higher at pH 7.5 than at pH 4.6. At pH 6.5 and above, *P*_*ZRT1*_-GFP expression was significantly higher than at pH 4.6.

In contrast, *P*_*ZRT2*_-GFP expression was not as strongly affected by the pH of the surrounding media, with expression at pH 4.6 being only 2-fold higher than at pH 6.5. These data are in agreement with the previous study of Bensen *et al*. who reported alkaline- and acidic- induction of *ZRT1* and *ZRT2*, respectively [28]. However, from our own observations, we conclude that expression of *ZRT1* is more strongly influenced by environmental pH than *ZRT2*.

These expression data support our hypothesis that Zrt2 is essential in acidic environments, whilst either Zrt1 or Zrt2 can support growth at neutral pH. To test this directly we created a *zrt1Δ*/*zrt2Δ* double mutant and performed more detailed growth kinetics analysis. **Fig 1C** shows that *zrt2Δ* again grew at neutral, but not acidic pH, whilst *zrt1Δ*/*zrt2Δ* failed to grow at both pH values. Growth was fully restored in the revertant strain (**Fig 1C**).

**Fig 2:**
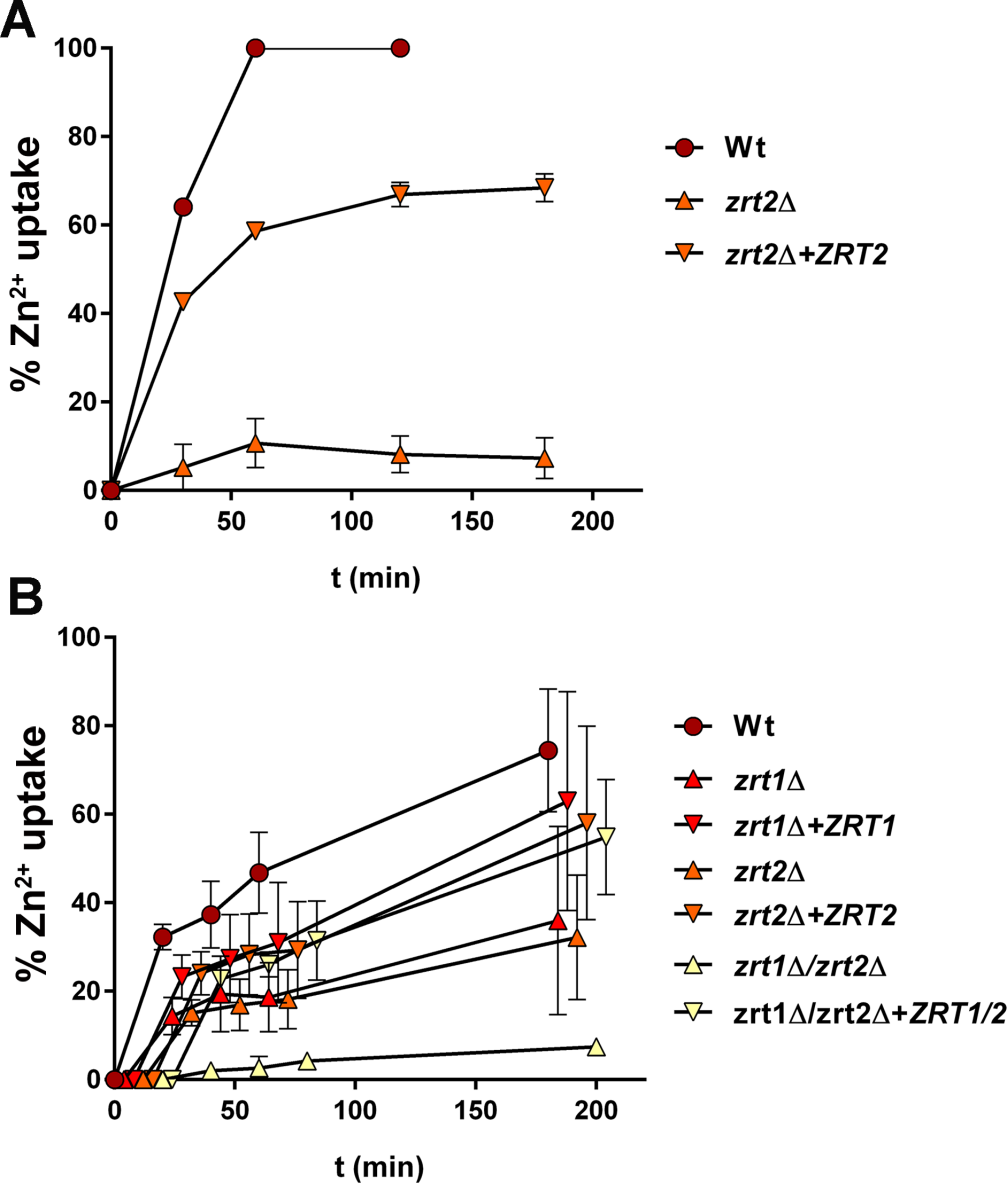
Zinc uptake by *C*. *albicans* is mediated by Zrt1 and Zrt2. (**A**) Indicated strains were cultured in low zinc medium (SD0, pH ~4.7), exposed to 25 μM ZnSO_4_ and zinc acquisition determined at indicated time points by measuring how much zinc remained in the cell free supernatant. *C*. *albicans* wild type acquires all measurable zinc within 60 minute; *zrt2*Δ does not; complementation restored zinc acquisition to 68%. Experiment performed three times (**B**) Indicated strains were incubated in RPMI without zinc for 24 h, exposed to 25 μM ZnSO_4_ and zinc acquisition determined as in panel A. Wild type cells acquire 74% of zinc by three hours; uptake is reduced by approximately 50% in *zrt1*Δ and *zrt2*Δ. *zrt1*Δ/*zrt2*Δ fails to take up zinc. Experiment performed twice. Data points have been shifted to the right to make them visible amongst strains.

The above growth and expression assays indicated that Zrt2 is the dominant cellular zinc transporter in *C*. *albicans* and the only functional importer at acidic pH. To test this, wild type, *zrt2Δ* and *zrt2Δ*+*ZRT2* were cultured in low zinc medium (SD zinc-dropout, acidic), provided with 25 μM Zn^++^ and zinc uptake from the medium measured. Wild type *C*. *albicans* sequestered all measurable zinc within 60 minutes. Zinc uptake was virtually abolished in the *zrt2Δ* mutant and *ZRT2* complementation restored uptake to 68% (**Fig 2A**). Therefore Zrt2 is essential for zinc acquisition by yeast cells in SD minimal medium.

We next assessed the relative impact of Zrt1 and Zrt2 on zinc uptake at neutral pH in RPMI medium (pH 8.2) at 37°C in tissue culture plates. Under these conditions the wild type took up 74% of zinc from the medium by 180 min (**Fig 2B**). In line with our observations that *ZRT1* and *ZRT2* are expressed at neutral pH, both *zrt1*Δ and *zrt2*Δ mutants acquired zinc from the medium, but this was reduced by approximately 50% compared to the wild type. Simultaneous deletion of both *ZRT1* and *ZRT2* abolished zinc uptake. Respective complementation with *ZRT1* and/or *ZRT2* increased zinc uptake to 55–63%. Therefore, both Zrt1 and Zrt2 contribute to zinc acquisition in RPMI.

In summary, Zrt2 is the major zinc importer in *C*. *albicans* whilst Zrt1 can support zinc uptake and growth specifically at neutral/alkaline pH.

**Fig 3:**
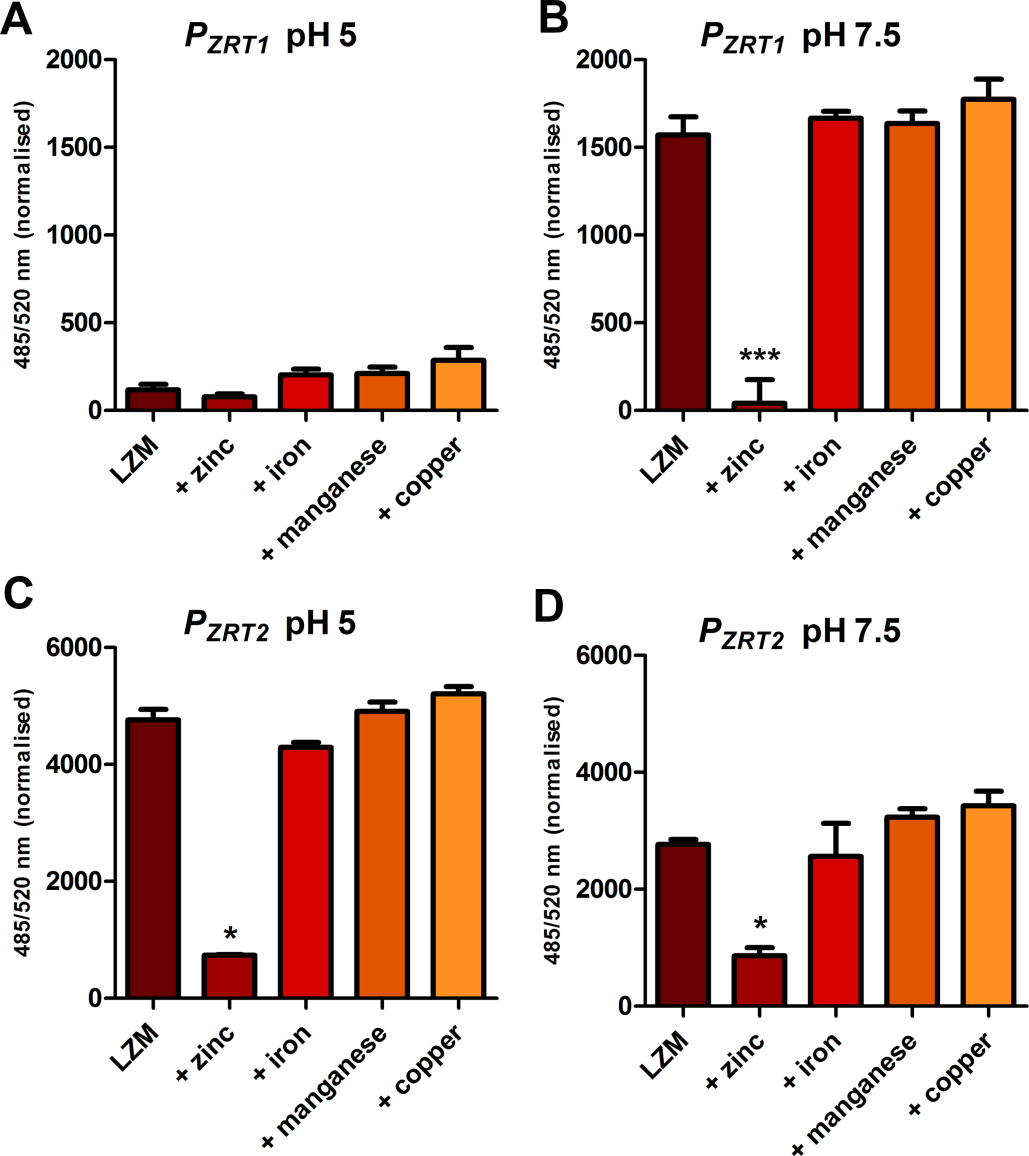
*P*_*ZRT1*_ and *P*_*ZRT2*_ metallo-regulation is zinc specific. Excess (100 μM) zinc, but not iron, manganese or copper downregulate *P*_*ZRT1*_-GFP (**A** and **B**) and *P*_*ZRT2*_-GFP (**C** and **D**). Experiment was performed three times. *(P <0.05) and *** (P <0.0001) = significantly different from LZM, Student’s t-test.

Both transporters are members of the Zip (Zrt/Irt protein) family, which also include iron transporters. Therefore, to assess the metal specificity of *ZRT1* and *ZRT2* regulation, we tested their expression in response to zinc and three other physiologically relevant trace metals—iron, manganese and copper. The reporter strains were incubated in low zinc media, buffered to pH 5 or to pH 7.5, and supplemented with zinc, iron, manganese or copper at 100 μM. At pH 5, *P*_*ZRT1*_ activity was again very low and supplementation with the different metals had no appreciable effect on expression (**Fig 3A**). At pH 7.5, *P*_*ZRT1*_ was 13.4-fold induced compared to pH 5 (**Fig 3A*vs*. 3B**). The addition of zinc to the medium resulted in 40-fold repression of *P*_*ZRT1*_ whilst iron, manganese and copper supplementation had no effect (**Fig 3B**).

*P*_*ZRT2*_ was again active in both acidic and neutral/alkaline media. At pH 5 and pH 7.5, zinc supplementation resulted in 6.5- and 3.2- fold repression, respectively. Supplementation with iron, manganese or copper had no effect (**Fig 3C & 3D**). From these data, we conclude that the metallo-regulation of *ZRT1* and *ZRT2* is zinc-specific in *C*. *albicans*.

**Fig 4:**
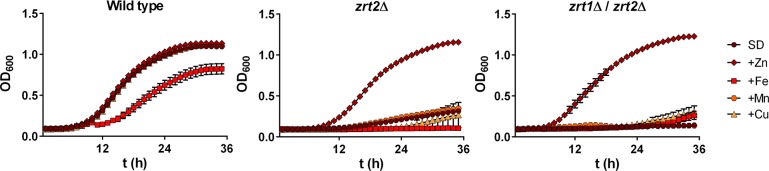
Growth of *zrt2*Δ strains is specifically rescued by excess zinc. Indicated strains were cultured as in Fig 1 with zinc, iron, manganese (100 μM) or copper (10 μM) and growth kinetics measured over 36 h in a microtitre plate. Experiment performed twice in triplicate. Iron had a moderate inhibitory effect on *C*. *albicans* growth. Note that only zinc rescued growth of *zrt2*Δ strains.

In order to functionally assess metal specificity, wild type, *zrt2*Δ and *zrt1*Δ/*zrt2*Δ cells were again cultured in minimal media, supplemented with zinc, iron, manganese, or copper. **Fig 4** shows that zinc, but not iron, manganese or copper supplementation restored growth, indicating that the growth defect of these mutants is due to an inability to acquire zinc in minimal media.

From these *in vitro* assays, it would appear that zinc transport in *C*. *albicans* is actually more similar to *A*. *fumigatus* than to *S*. *cerevisiae*. Baker’s yeast encodes two plasma membrane importers: the high affinity Zrt1 and low affinity Zrt2, neither of which are known to be subject to pH-regulation [8,9]. In contrast, *A*. *fumigatus* encodes three zinc importers: ZrfA and ZrfB, which are expressed in acidic environments, and ZrfC, which is expressed at neutral/alkaline [25,27]. As *A*. *fumigatus* ZrfB and ZrfC are respective orthologues of *C*. *albicans* Zrt2 and Zrt1 [30], this suggests that zinc transporter pH-dependence may be conserved in multiple fungal species. In [30] and in supplementary data **S4 Fig** we propose an evolutionary framework of how pH adaptation may have shaped the evolution of fungal zinc transporters.

### Zinc uptake during invasive candidiasis

**Fig 5:**
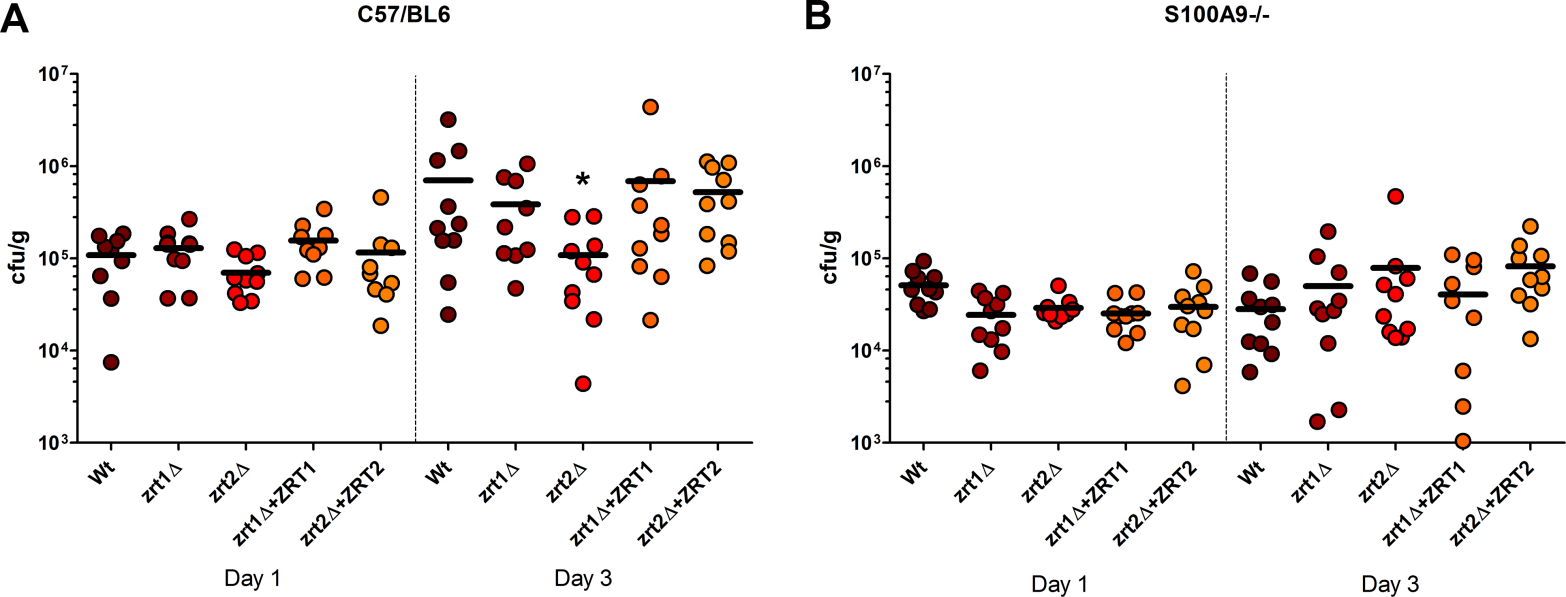
*C*. *albicans* Zrt2 is required for kidney colonisation in the presence of functional calprotectin. Indicated mice strains were infected with indicated fungal strains and kidney colonisation determined by plating CFUs on day one and day three post-infection. At day three post-infection, *C*. *albicans* wild type kidney fungal burden had increased significantly by 6.5-fold (*P* = 0.034), Deletion of *ZRT2* precluded an increase in kidney fungal burden between day one and day three post-infection (*P* = 0.597), asterisk. Complementation of *zrt2*Δ with a single copy of *ZRT2* restored kidney colonisation at day three (4.5-fold higher than at day one, *P* = 0.004).

The role of zinc uptake in *C*. *albicans* virulence remains largely unexplored. Here we used a murine model of disseminated candidiasis to directly assess the role of Zrt1 and Zrt2 in *C*. *albicans* fitness *in vivo*. Mice were infected intravenously and kidney fungal burden assessed at day one and day three post-infection. **Fig 5A** shows that by day one post-infection, all strains exhibited similar levels of kidney fungal burden, indicating that neither Zrt1 nor Zrt2 are required for initial kidney colonisation.

However, by day three post-infection, *C*. *albicans* wild type kidney fungal burden had increased significantly by 6.5-fold (*P* = 0.034), indicating that cells had proliferated in this organ. In contrast, deletion of *ZRT2* precluded an increase in kidney fungal burden between day one and day three post-infection (*P* = 0.597). Complementation of *zrt2*Δ with a single copy of *ZRT2* restored kidney colonisation at day three (4.5-fold higher than at day one, *P* = 0.004). In contrast, deletion of *ZRT1* did not inhibit fungal proliferation in the kidney. These data indicate that Zrt1 and Zrt2 are dispensable for initial kidney colonisation (day one post-infection) and that Zrt2 is important for systemic candidiasis at later stages. These data are in agreement with previous studies. Xu and co-workers identified a transcription factor, Sut1, which governs the expression of zinc assimilation genes during invasive candidiasis [31]. Deletion of *SUT1* attenuated *C*. *albicans* virulence, however, *sut1*Δ virulence was restored to wild type levels via *ZRT2* overexpression, indicating that defective *in vivo* expression of *ZRT2* was responsible for the attenuated virulence of *sut1*Δ [31]. Several other studies have analysed the *C*. *albicans* transcriptome during kidney colonisation. Walker and co-workers reported that only two genes were transcriptionally upregulated during both rabbit [32] and mouse [33] kidney colonisation: *ADR1* and *ZRT2*. *ZRT2* is also upregulated during *in vitro* incubation with macrophages [34]. Combined with the *zrt2*Δ *in vivo* growth defect reported here, these expression studies suggest an important role for Zrt2 in zinc uptake during invasive candidiasis.

We next addressed the role of host-driven nutritional immunity on fungal growth *in vivo*. Calprotectin plays a key role in mediating zinc nutritional immunity. Calprotectin is a heterodimeric protein composed of S100A8 and S100A9 subunits which has potent antifungal activity via zinc sequestration [35,36]. Calprotectin expression in *C*. *albicans*-infected murine kidney tissue has been reported to be upregulated between day one and day three post-infection in two independent studies [36,37]. As Zrt2 is important for growth under zinc limitation *in vitro* and exhibited impaired growth *in vivo*, we examined the impact of calprotectin on *C*. *albicans* kidney colonisation. Surprisingly, all five tested *C*. *albicans* strains exhibited lower kidney fungal burdens in calprotectin-deficient mice than in wild type animals at both day one and day three post-infection (**Fig 5B**). At day three post-infection, the fungal burden of calprotectin-deficient mouse kidneys infected with wild type *C*. *albicans* was significantly lower (p < 0.05) than wild type mice infected with the same strain. This was unexpected, as calprotectin-deficient mice have been previously shown to succumb earlier to *C*. *albicans* infections [35], however a recent study has also reported lower kidney fungal burden in calprotectin deficient mice compared to wild type [36]. In addition to its anti-fungal activity via zinc sequestration, calprotectin plays additional roles in immunity. Indeed, as well as its role in nutritional immunity, calprotectin has been implicated as an inflammatory mediator and has been shown to exacerbate disease in other models of candidiasis [38]. These additional immune properties may explain the decreased fungal burden observed in calprotectin-deficient mice. Nevertheless, in calprotectin-deficient mice, the *zrt2*Δ mutant did not exhibit a notable difference in kidney colonisation compared to wild type *C*. *albicans*. This indicates that, in the absence of a host calprotectin response, fungal Zrt2 is dispensable for kidney colonisation by *C*. *albicans*.

Calprotectin constitutes around half the cytoplasmic protein content of neutrophils and is a major component of neutrophil extracellular traps (NETs), from which it elicits its antifungal activity via zinc sequestration [35,39]. In order to explore the host-pathogen relationship between pathogen Zrt2 and host calprotectin in greater detail, we next compared the antifungal properties of wild type and calprotectin-deficient NETs.

**Fig 6:**
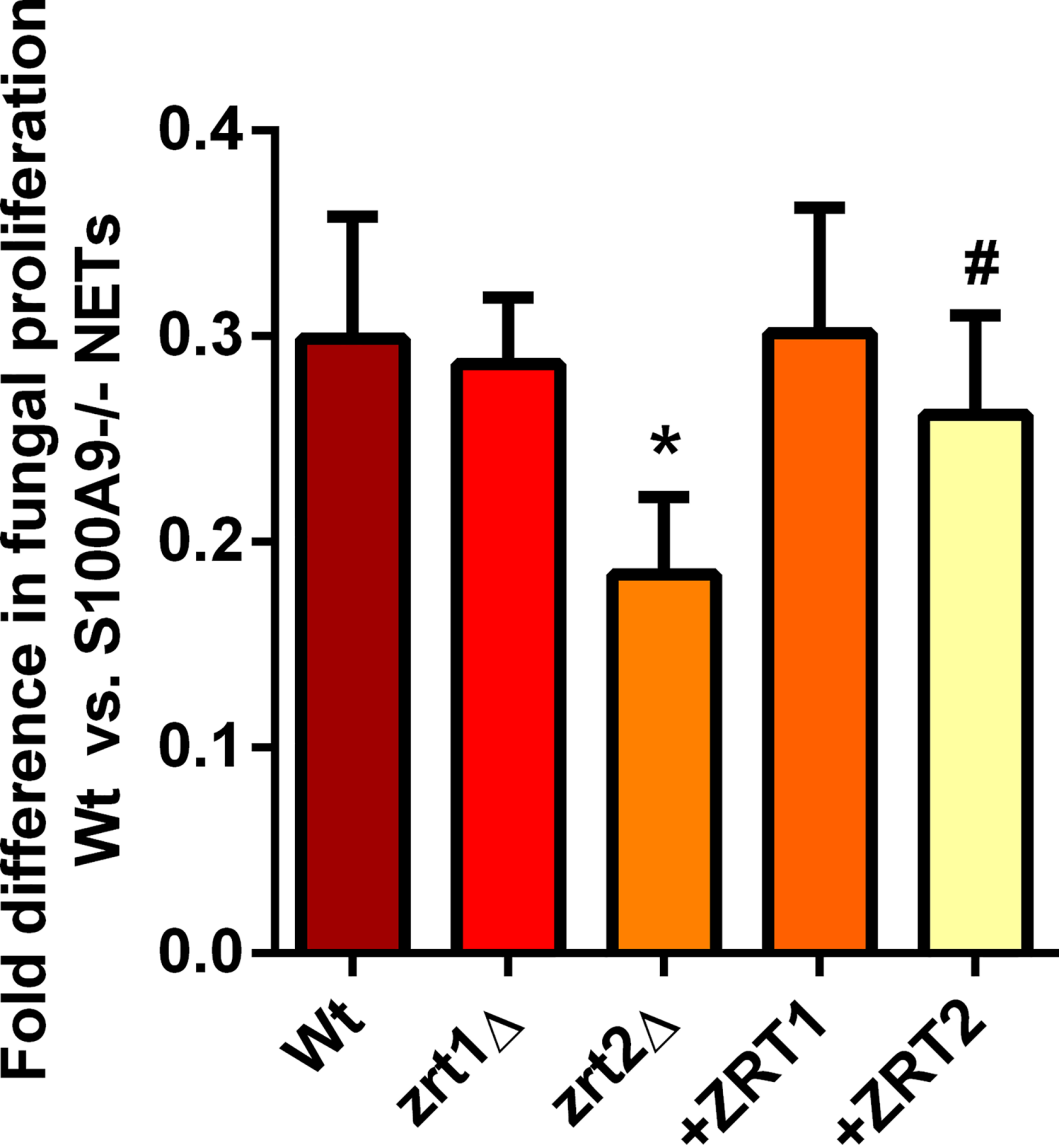
Zrt2 protects against calprotectin-dependent inhibition of fungal growth during *C*. *albicans*-neutrophil extracellular trap interaction. Indicated strains were incubated with wild type or S100A9-/- -derived NETs or in medium only. Following ~21 hours incubation, metabolic activity was determined by XTT assay. Activity in the presence of both NET groups was determined compared to control conditions in the absence of NETs. Experiment was performed three time. * indicates P < 0.05 and # not significantly different to wild type, Students t-test Data presented are fold reduction in activity due to the presence of calprotectin.

Calprotectin-decoration of NETs and associated antifungal activity via zinc sequestration has been well defined [35]. In line with this, S100A9-/- NETs exhibited highly attenuated antifungal activity compared to NETs from wild type neutrophils (**S6 Fig**). Deletion of *ZRT2* rendered *C*. *albicans* sensitive to NET antifungal activity in a calprotectin-dependent manner (**Fig 6**), suggesting a role for Zrt2 in growth in the presence of calprotectin^+^ NETs. In summary, host (calprotectin) and pathogen (Zrt2) factors appear to define the struggle for zinc during *C*. *albicans* infection: Zrt2 is the major zinc transporter of this important fungal pathogen and is essential for growth in the presence of calprotectin *in vivo* and *ex vivo*.

From this study, and work from the groups of Mitchell, Calera, Deepe, Staats and Jung, it is becoming increasing clear that zinc acquisition plays a critical role in fungal pathogenesis, as perturbation of zinc transporter function in *C*. *albicans*, *A*. *fumigatus*, *H*. *capsulatum*, *C*. *neoformans* and *C*. *gattii* attenuates virulence in all five organisms tested thus far [14–17]. Moreover, deletion of the master regulator gene of zinc homeostasis in fungi, *ZAP1*, also attenuates virulence in *A*. *fumigatus*, *C*. *gattii* and *C*. *dubliniensis* and decreases *in vivo* fitness in *C*. *albicans* [40–43]. In supplementary information **S5 Fig** we discuss how different zinc uptake genes are differentially required for virulence in the major fungal pathogens of humans.

### The role of intracellular compartmentalisation in adaptation to environmental zinc

We next sought to address how the fungal cell deals with zinc following its internalisation. This is an important issue because, as well as serving as an essential micronutrient, zinc can be highly toxic to cells. In order to assess the dynamics of intracellular zinc compartmentalisation, we utilised zinquin. Zinquin is a zinc-specific fluorescent probe which accumulates in storage vesicles known as zincosomes and fluoresces upon zinc binding [44].

**Fig 7:**
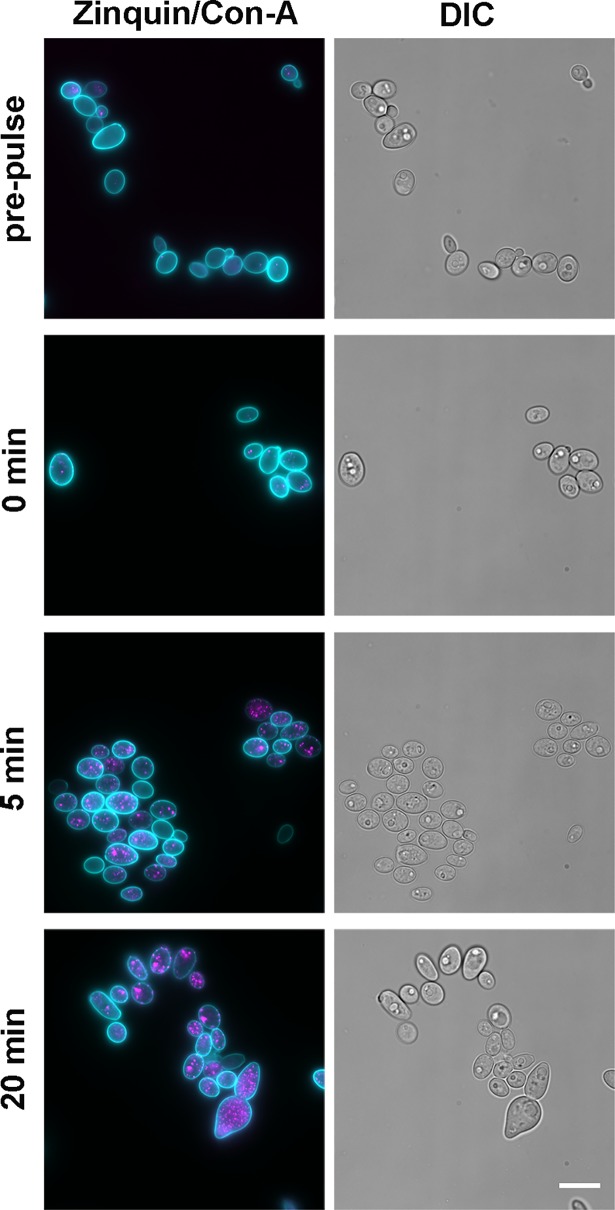
Kinetics of zincosome formation in *C*. *albicans*. Cells were incubated overnight in YNB-zinc-dropout medium (SD0) to deplete zincosomes and pulsed with 25 μM ZnSO_4_ for indicated time points. Cells were then stained with zinquin to probe for zincosomal zinc and the cell wall stained with Concanavalin A conjugated to Alexa-647. Left hand column shows false colour overlay of cell wall (cyan) and zincosomes (magenta). Right hand column shows DIC; Experiment performed three times and representative images shown.

Zinc-depleted cells, prepared by growing the cells overnight in low zinc medium, were pulsed with 25 μM zinc, washed and fixed at five minute intervals and stained with zinquin. **Fig 7** shows that even with immediate washing and fixation, *C*. *albicans* already stained positive with zinquin, indicating that zincosomal zinc compartmentalisation upon exposure to zinc occurs rapidly. By 20 minutes post-pulse, the majority of cells exhibited numerous zincosomes as indicated by zinquin fluorescence. Therefore, *C*. *albicans* rapidly compartmentalises zinc within zincosomes in response to changes in environmental zinc.

We therefore turned our attention to ZnT-type transporters which, in contrast to Zip transporters (such as Zrt2), transport their substrate from the cytoplasm to either outside the cell, or into the lumen of intracellular compartments [7].

Five *C*. *albicans* ZnT-type (PF01545) transporters were identified using FungiDB with sequence similarity to *S*. *cerevisiae* Mmt1/2 (orf19.52), Zrg17 (orf19.3769), Msc2 (orf19.3132), and Cot1/Zrc1 (orf19.1536), as well as a fifth protein encoded by orf19.3874 which does not have an orthologue in *S*. *cerevisiae* (**Table 1**). We therefore created deletion mutants for these five putative zinc transporter genes. For orf19.1536, we propose the common name, Zrc1.

**Table 1:** Identified ZnT-type transporter in *C*. *albicans* and their relationship with *S*. *cerevisiae*.

orf19.	Yeast best hit	E value	Yeast description
orf19.1536	Zrc1 / Cot1	2.6e-94 / 5.3e-90	Vacuolar zinc importer
orf19.3874	None		
orf19.3769	Zrg17	1.5e-39	ER zinc import (heterodimer with MSc2)
orf19.3132	Msc2 (/ Zrc1 partial)	3e-76 (/ 1.4e-28)	ER zinc import (heterodimer with Zrg17)
orf19.52	Mmt2/1	3.3e-62 / 3.9e-60	Mitochondrial iron import

To determine which ZnT-transporter may mediate zincosome compartmentalisation, wild type, *zrc1*Δ, orf19.3874Δ, orf19.3769Δ, orf19.3132Δ, and orf19.52Δ cells were pulsed with zinc for 20 minutes and stained with zinquin.

**Fig 8:**
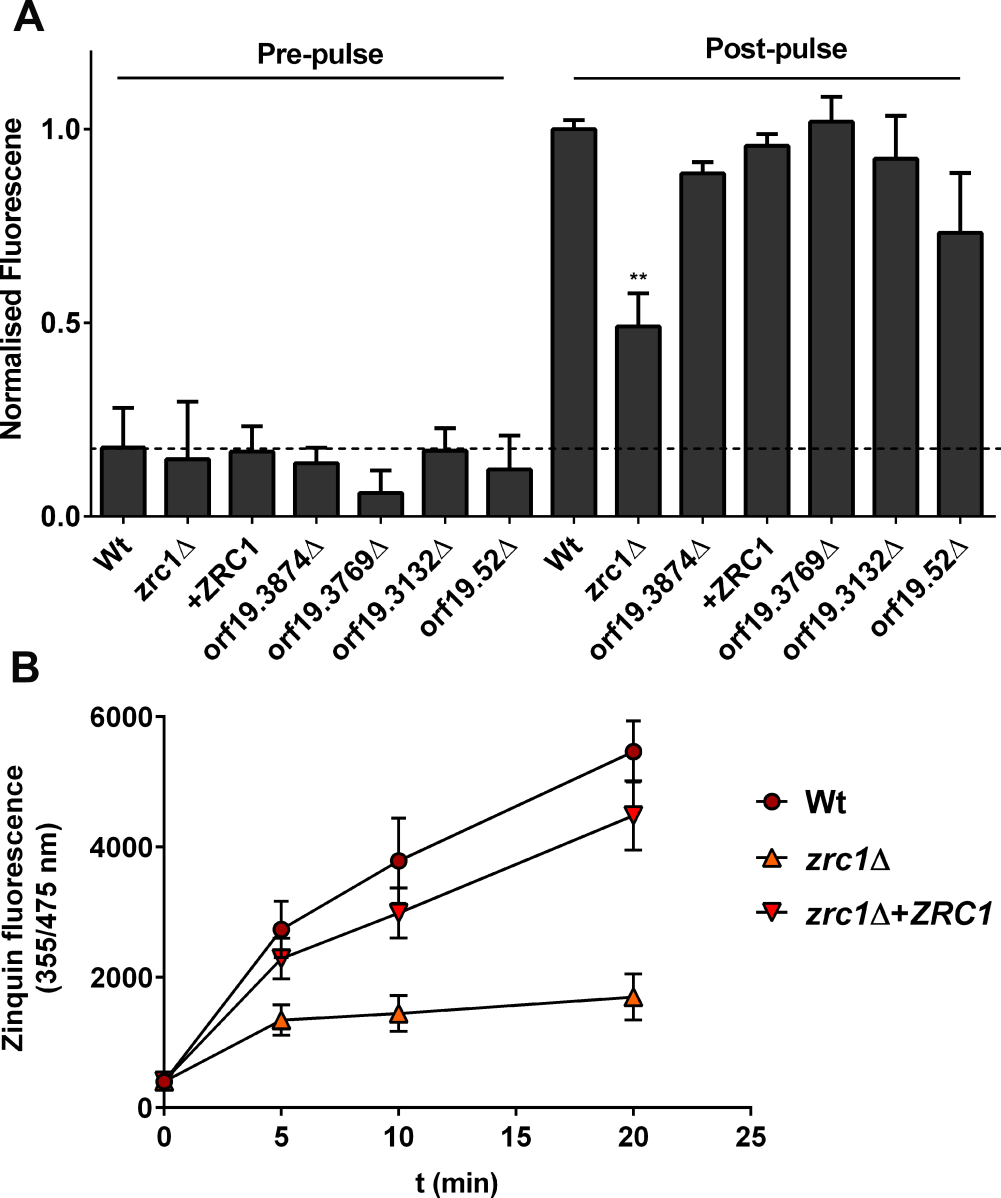
Zincosome formation is Zrc1 dependent. (A) Zincosome screen. Wild type, ZnT deletion mutants and *zrc1*Δ+*ZRC1* strains were pulsed with 25 μM zinc for 20 minutes and zincosome fluorescence determined by staining with zinquin. Prepulsed cells were also stained as control. Experiment was performed at least twice in duplicates and all data normalised to the post-pulse value of wild type. ANOVA was first performed on initial (pre-normalised data). Asterisks indicate statistical significance compared to wild type and to relevant deletion mutant ** P <0.01. (B) As panel A, except zinquin fluorescence kinetics was determined by flow cytometry. Experiment performed three times. *zrc1*Δ exhibits significantly reduced zinquin fluorescence compared to wild type and revertant at 20 minutes *P* < 0.001, ANOVA.

**Fig 8A** shows that the isogenic wild type exhibited a significant 5.6-fold increase in zinquin fluorescence following the zinc pulse Deletion of orf19.3874, orf19.3769 or orf19.3132 had no effect in this assay. The orf19.52Δ mutant exhibited perturbed zincosome generation, but this was not significant under the conditions tested here. Deletion of *ZRC1*, on the other hand, strongly inhibited zincosome formation and this was restored to wild type levels by genetic complementation with a single copy of *ZRC1* (**Fig 8A**).

This screen indicated that the ZnT-type transporter, Zrc1, plays a role in zincosome formation. For wild type, *zrc1*Δ and *zrc1*Δ+*ZRC1* strains, the experiment was repeated and zincosome accumulation determined at 5, 10 and 20 minutes post-pulse by flow cytometry. **Fig 8B** shows that both wild type and *zrc1*Δ+*ZRC1* strains exhibited progressive increases in zinquin fluorescence following the zinc pulse, resulting in more than a 10-fold increase by 20 minutes compared to the pre-pulse condition. In contrast, *zrc1*Δ exhibited only a moderate (~3-fold) increase in fluorescence by 5 minutes, and the signal did not significantly increase at later time points. These data show that the ZnT-type transporter Zrc1 is required for zincosomal zinc accumulation in *C*. *albicans*. Interestingly, when we measured actual zinc uptake within this shorter time period, cells took up less than 30% within 20 minutes, suggesting that these very early zincosome formation events (**Figs 7 and 8**) may be the result of intracellular zinc mobilisation, prior to significant cellular uptake (**Fig 2**). Indeed, we have very recently demonstrated that *C*. *albicans* undergoes very rapid (seconds) remobilisation of intracellular zinc pools upon changes in environmental zinc, in the absence of cellular uptake [45].

The kinetics of zincosome formation in the model yeast *S*. *cerevisiae* have been reported to be similar to those described here, occurring within 5–20 minutes exposure of zinc-depleted cells to a zinc pulse [12]. However, the mechanistic basis of zincosomal zinc accumulation appears to be fundamentally different in these two species. *S*. *cerevisiae* encodes two orthologues of *C*. *albicans* Zrc1: Zrc1 and Cot1. However, single *zrc1*Δ, *cot1*Δ and *zrc1*Δ/*cot1*Δ double mutants exhibited wild type zincosome formation, suggesting that neither ScZrc1 nor its paralogue, Cot1, are involved in zincosome formation in *S*. *cerevisiae* [12]. In fact, *S*. *cerevisiae* Zrc1 instead plays a clear and important role in vacuolar zinc accumulation [11].

**Fig 9:**
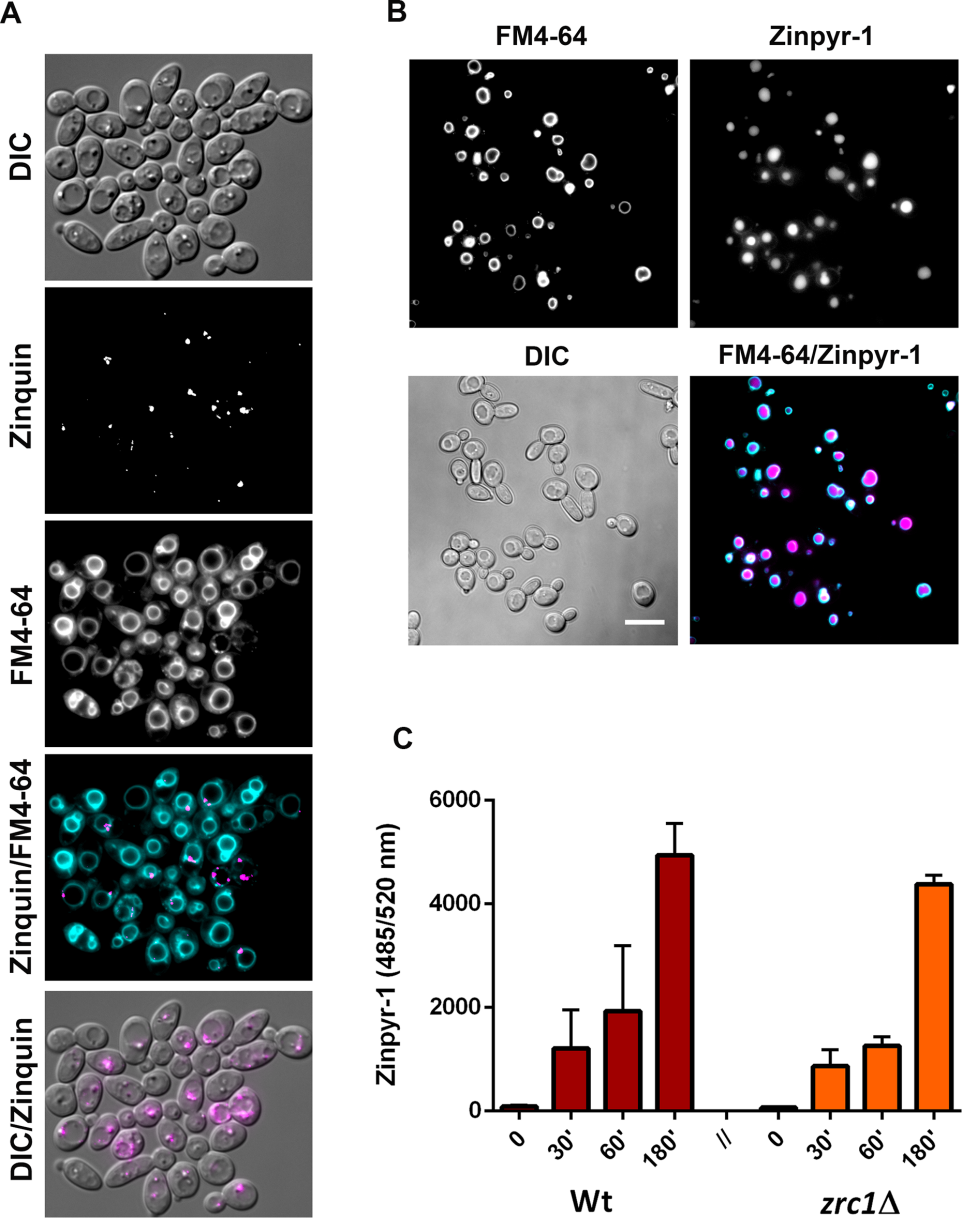
Relationship between zincosomes and vacuole in *C*. *albicans*. (**A**) Cells were co-stained with zinquin (zincosomes) and FM4-64, which stains the fungal vacuole membrane. Note that zincosomes are not intra-vacuolar. (**B**) The zinc-specific probe Zinpyr-1 can be used to detect vacuolar zinc in *C*. *albicans*. Cells were co-stained with Zinpyr-1 and FM4-64. Note that Zinpyr-1 stains vacuolar zinc in *C*. *albicans* (**C**) Zrc1 is not required for vacuolar zinc import. Cells were loaded with Zinpyr-1, pulsed with 25 μM zinc and Zinpyr-1 fluorescence determined at 0, 30, 60 and 180 minutes post pulse. Experiments performed at least twice.

We therefore sought to characterise the relationship between our novel Zrc1-zincosome pathway and vacuolar zinc in *C*. *albicans*. Co-staining cells with zinquin and the vacuolar membrane dye FM4-64 [46] revealed that zincosomes are not found within the fungal vacuole in *C*. *albicans* but rather, close to the outer leaflet of the vacuolar membrane (**Fig 9A**). Given this relatively close spatial relationship, we next questioned whether Zrc1-dependent zincosomal zinc compartmentalisation was an upstream component of vacuolar zinc trafficking in *C*. *albicans*. We first established that *C*. *albicans* can sequester zinc within the vacuole using the fluorescent probe Zinpyr1 (**Fig 9B**). Interestingly, in our zinc-pulse experiment, *zrc1*Δ accumulated vacuolar zinc to the same levels as the wild type, even after extended incubation (**Fig 9C**). Therefore, under the conditions tested, Zrc1 in *C*. *albicans* is not essential for vacuolar zinc import.

**Fig 10:**
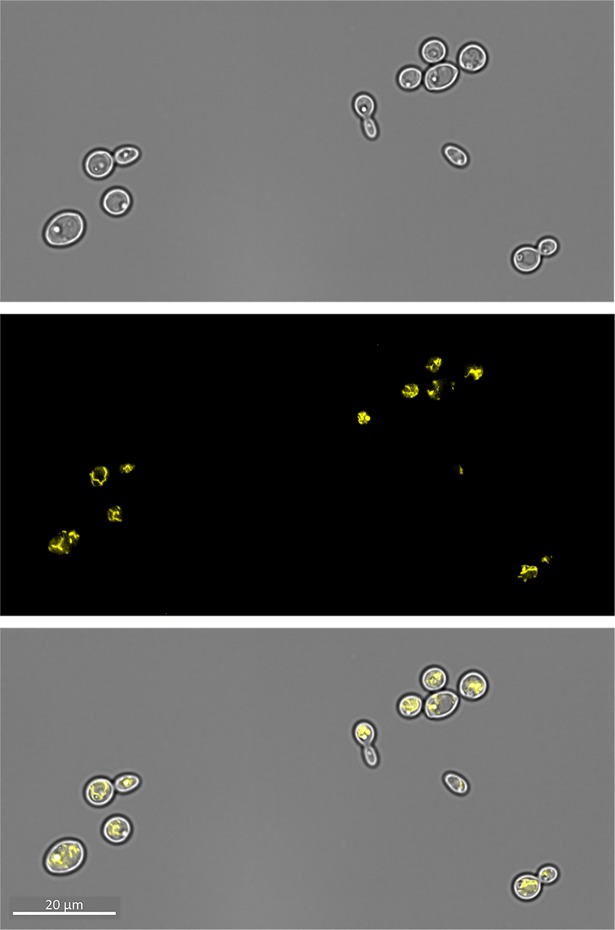
Zrc1 exhibits intracellular membrane localisation. The remaining copy of Zrc1 in a *zrc1*Δ/*ZRC1* heterozygous mutant was tagged at its C-terminus with a codon optimised Venus yellow fluorescent protein. The resulting strain was incubated for 24 h in SD0, treated with 25 μM zinc for 20 minutes and imaged. Note that Zrc1 does not localise exclusively to the vacuole as is the case in *S*. *cerevisiae* and *C*. *neoformans*, but rather to the internal membrane system, reminiscent of the endoplasmic reticulum. Experiment was performed twice.

Zrc1 has been reported to localise to the vacuole in *S*. *cerevisiae* and *C*. *neoformans* [47,48]. However, our own analysis indicated that *C*. *albicans* Zrc1 is dispensable for vacuolar zinc import under the conditions tested here (**Fig 9C**). To test whether Zrc1 localises to the *C*. *albicans* vacuole, we tagged the protein at its C-terminus with a codon optimised Venus fluorescent protein. **Fig 10** shows that *C*. *albicans* Zrc1, unlike its *S*. *cerevisiae* and *C*. *neoformans* orthologues, does not localise predominantly to the vacuolar membrane, but instead to the internal membrane system, reminiscent of the endoplasmic reticulum. This localisation is more similar to that of *Schizosaccharomyces pombe* Zhf1 which transports zinc into the endoplasmic reticulum [49].

### Zrc1-dependent zincosomal detoxification is essential for adaptation to environmental zinc

Given the importance of Zrc1-mediated vacuolar zinc detoxification in the model yeast *S*. *cerevisiae* and in the basidiomycete pathogen *C*. *neoformans*, we next questioned whether a relationship exists between Zrc1, zincosomes, and metal tolerance in *C*. *albicans*.

First, we screened *zrc1*Δ, as well as all other ZnT-transporter deficient mutants for sensitivity to log_10_-fold increases in Zn^++^, Fe^++^, Mn^++^ and Cu^++^ alone or in combination. We included the other mutant strains and other metals to test for potential redundancy and transporter promiscuity. We did not observe significant synergistic toxicity of the tested metals, however excess manganese protected cells from zinc toxicity. The mutant lacking orf19.3874 exhibited increased sensitivity to excess manganese and all strains exhibited relatively similar levels of iron and copper tolerance. (**S7 Fig**).

**Fig 11:**
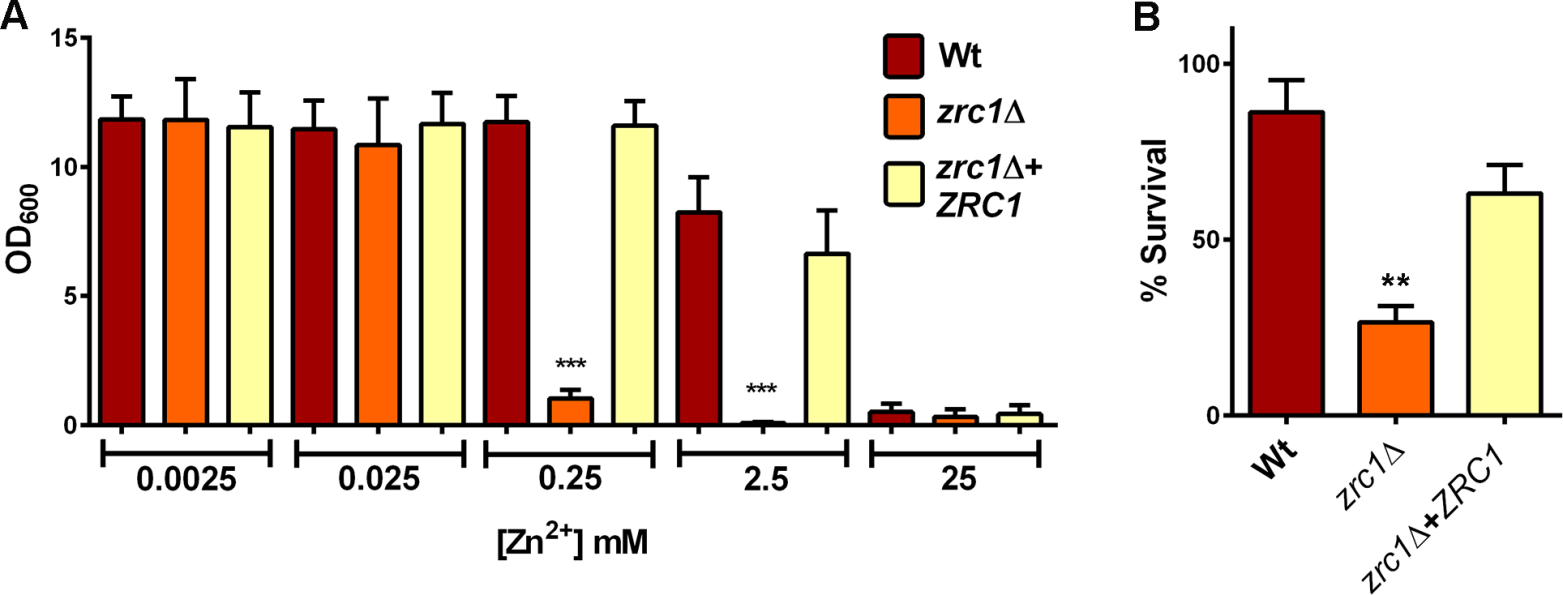
Zrc1 is essential for zinc detoxification. (**A**) Strains were cultured for 24 h in SD0 medium containing indicated zinc supplementation. Experiment performed at least three times in duplicate for zinc concentrations at 25 μM and above. *** indicates significant difference (*P* < 0.001) compared to wild type and revertant, ANOVA. (**B**) Strains were precultured in SD0, challenged with 1 M ZnSO_4_ for 3 h and viability assessed by measuring CFUs. ** indicates significant difference (*P* < 0.01) compared to wild type and revertant, ANOVA. Experiment performed three times for wild type and *zrc1*Δ and twice in duplicate for all three strains.

Lack of Zrc1, on the other hand, resulted in approximately 100-fold increased Zn^++^-sensitivity (**Fig 11A and S7 Fig**) and genetic complementation restored Zn^++^ tolerance back to wild type levels (**Fig 11A**). The observed Zn^++^ sensitivity of *C*. *albicans* observed in these experiments is likely due growth inhibition, rather than fungal killing. Indeed, we had to expose cells to molar concentrations of zinc to kill *C*. *albicans*. Although *zrc1*Δ was also hypersensitive to Zn^++^ killing (**Fig 11B**), it is unclear whether *C*. *albicans* will face such high levels of Zn^++^ in nature. On the other hand, sub-millimolar to millimolar concentrations are well within the physiological range *C*. *albicans* will likely face in its natural environment as a human commensal and pathogen. Therefore, *C*. *albicans* Zrc1 plays a crucial role in adaptation to environmental zinc.

**Fig 12:**
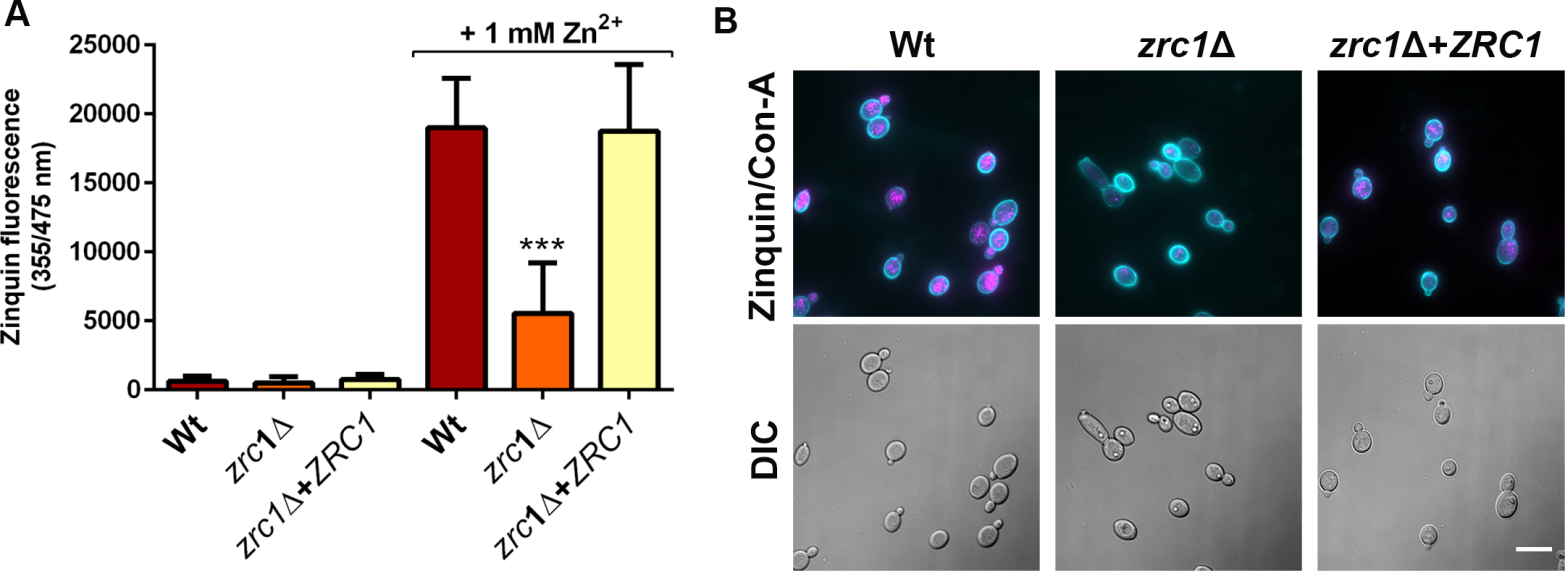
Relationship between Zrc1, zincosomes and zinc tolerance. (A) Cells were challenged with potentially toxic zinc (1 mM), stained with zinquin and fluorescence determined. *P* < 0.0001 compared to wild type and revertant. (B) Micrographs of cells treated as in A. Note that *zrc1*Δ is highly defective for zincosome formation in response to 1 mM ZnSO_4_ –a condition under which wild type, but not *zrc1*Δ cells can grow (S7 Fig).

To examine whether there was a link between Zrc1-dependent zinc tolerance and zincosome formation, we exposed cells to 1 mM Zn^++^ for 2 h and measured zinquin fluorescence. This was chosen because Zrc1 is essential for growth at this concentration (**S7 Fig and Fig 11A**) and, whilst it is tolerated by wild type cells, is close to toxicity. Wild type *C*. *albicans* cells exhibited a considerable (31-fold) increase in zinquin fluorescence in response to challenge with 1 mM Zn^++^. This was significantly reduced in *zrc1*Δ and restored to wild type levels by genetic complementation with *ZRC1* (**Fig 12A**). Fluorescence microscopy revealed that these quantitative measurements reflect zincosome formation in wild type and *zrc1*Δ+*ZRC1*, but not in *zrc1*Δ cells (**Fig 12B**). Therefore, Zrc1 plays a crucial role in zincosomal zinc compartmentalisation in response to both relatively minor fluctuations in zinc availability (**Fig 8**) and potentially toxic levels of heavy metal (**Figs 11A and 12**). Together these data suggest that Zrc1-dependent zincosome formation is important for *C*. *albicans* adaptation to environmental zinc.

**Fig 13:**
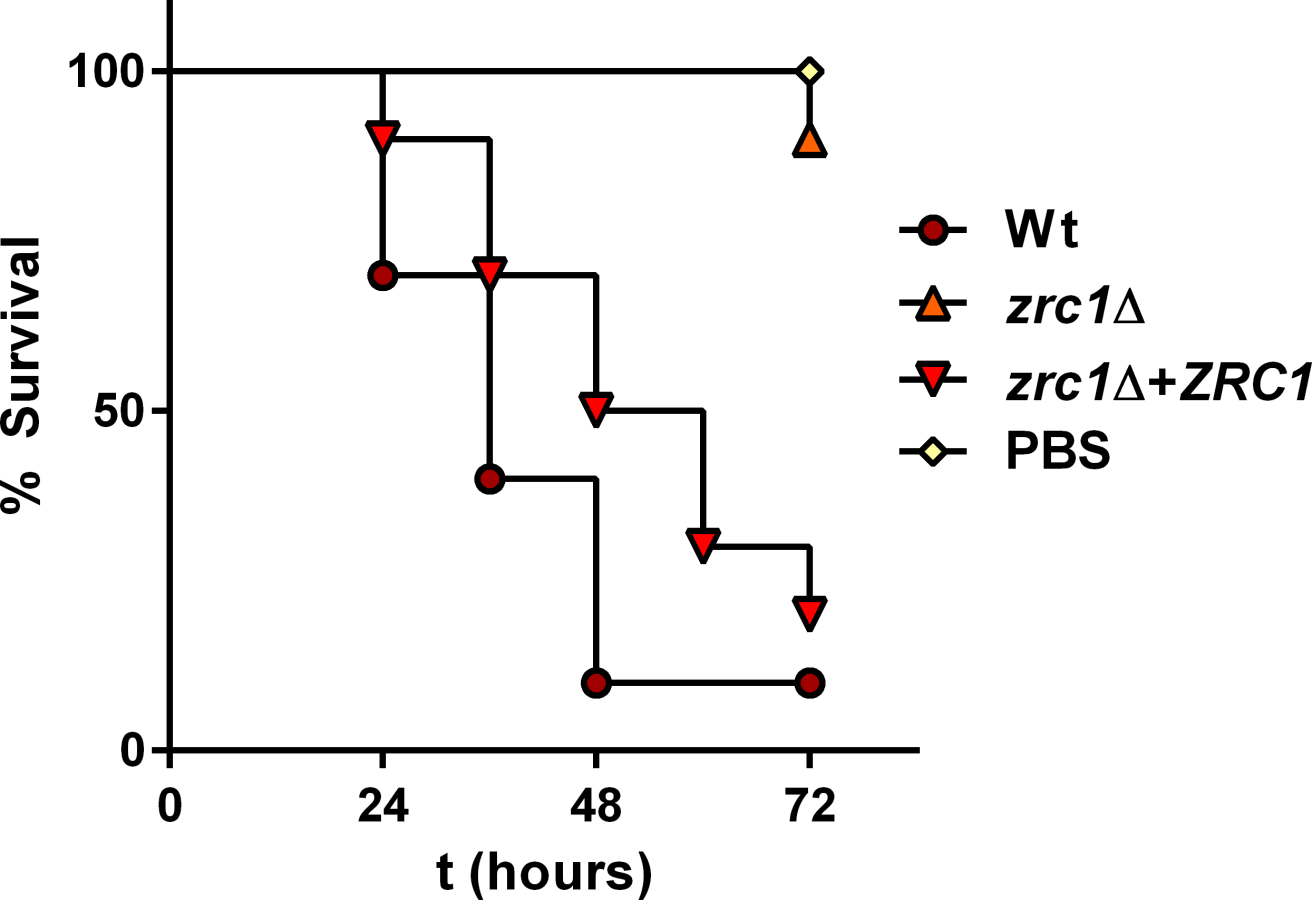
Zrc1 is required for virulence in a *Galleria* infection model. *Galleria* larvae (10 per group) were infected with 10^5^ *C*. *albicans* cells and monitored every 12 h. Note that whilst wild type result in high mortality, only one *zrc1*Δ-infected larvae died. Experiment performed twice—here, and in S8 Fig. *zrc1*Δ is significantly attenuated compared to wild type (P = 0.0001) and *zrc1*Δ+*ZRC1* (P = 0.0009), but not compared to PBS control (P = 0.3173); Log-rank (Mantel-Cox) test.

The current study is amongst the first detailed reports of intracellular zinc trafficking in a human fungal pathogen. We therefore assessed whether *C*. *albicans* Zrc1 plays a role in virulence. For this we chose two different infection models. Insect larvae have been reported to accumulate high levels of zinc [50]. We therefore first performed infection experiments on the commonly used *Galleria mellonella* larva. The majority of wild type and *zrc1*Δ+*ZRC1* infected larvae succumbed to infection within 2–3 days post infection. Strikingly, only a single *zrc1*Δ infected larvae died in these experiments, showing that Zrc1 is essential for virulence in this model (**Fig 13** & **S8 Fig**). Although *C*. *albicans* is not a known pathogen of insect larvae, this observation is interesting because it suggests that *Galleria* may possess a form of high-zinc nutritional immunity; a phenomenon which has been reported in mammals [5] and, recently, in plants [51].

**Fig 14:**
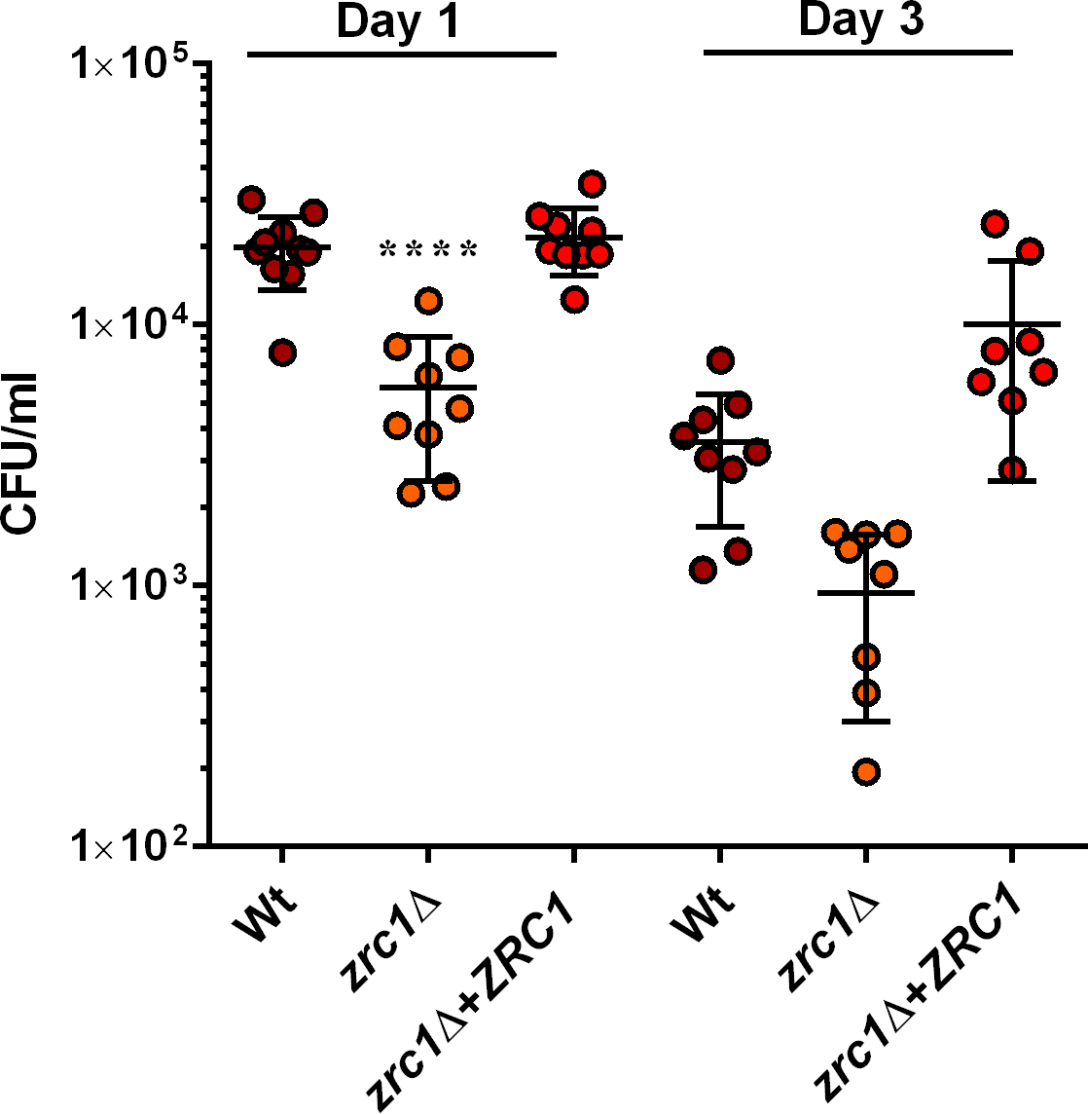
Zrc1 is essential for liver colonisation. Mice were infected with indicated fungal strains and liver colonisation determined by plating CFUs on day one and day three post-infection. Asterisks indicate significant difference compared to wild type and revertant, ANOVA.

In mammals, inflammation and the acute phase response result in zinc is trafficking to the liver in order to induce zincaemia [52]. We therefore assessed the capacity of *zrc1*Δ to colonise the murine liver. As shown in **Fig 14**, *zrc1*Δ exhibited a clear and significant defect in liver colonisation compared to both wild type and *zrc1*Δ+*ZRC1*. In contrast, *zrc1*Δ exhibited the same kidney fungal burden as the wild type (**S9 Fig**). *zrc1*Δ mice gained 5% body weight between day 1 and day 3 post infection, whilst wild type and *zrc1*Δ+*ZRC1* infected mice lost weight (1.2–3%). This, together with larval survival (**Fig 13**) and liver colonisation (**Fig 14**) data indicate that Zrc1 plays an important role in *C*. *albicans* virulence.

In summary, we have described a novel pathway of zinc import and compartmentalisation in *C*. *albicans* and demonstrated the significance of these mechanisms for both microbial physiology and *in vivo* fitness. Interestingly, the cellular import pathway of this fungus appears to be highly similar to that of *A*. *fumigatus* and we have proposed an ecological-evolutionary framework which may explain some of the conservation and divergence that we observe in extant human fungal pathogenic species. We also demonstrate that unlike any previously characterised pathogenic fungi, *C*. *albicans* assimilates zinc from environment to zincosomes using a Zrt1,2/Zrc1-dependent biphasic mechanism.

## Methods

### Strain construction

*C*. *albicans* strains used in this study are listed in **S1 Table**. The triple-auxotrophic strain BWP17 complemented with plasmid CIp30 served as the isogenic wild type control in all experiments. Homozygous *C*. *albicans* mutants were constructed as described previously [53] and the primers used for this are listed in **S1 Table**. Briefly, forward primers were designed with 104 bp homology to the immediate upstream region of the gene of interest, followed by a 22 bp sequence, with homology to the pFA plasmids, immediately upstream of the respective selective marker. Similarly, reverse primers were designed with 104 bp homology to the immediate downstream region of the gene of interest (reverse complement), followed by 24 bp sequence with homology to the pFA plasmids, downstream of the selective marker.

These long primers, together with plasmids pFA-HIS1 and pFA-ARG4 were used to create deletion constructs for each of the zinc transporter encoding genes and the two alleles of each gene sequentially deleted using the improved transformation protocol [54] and selecting for histidine or arginine prototrophy. In each case, correct integration was determined using gene-specific upstream and downstream primers, lying outside the site of homologous recombination to determine absence of wild type copy and presence of::*HIS1* and::*ARG4* alleles, as well as *HIS1* and *ARG4* specific internal primers to ensure correct integration of selective markers at both 5’ and 3’. For double deletion of *ZRT1* and *ZRT2*, the *zrt1*Δ uridine auxotrophy was sequentially transformed by the SAT flipper technique to delete both copies of *ZRT2*. All these uridine auxotrophs were *URA3* complemented with *Nco*I-linearised CIp10 plasmid [55]. For the double mutant, both *ZRT1* and *ZRT2* including up- and down- stream sequences were sub-cloned into CIp10. For *ZRT2* and *ZRC1*, the wild type alleles, together with the up- and down- stream intergenic regions were amplified from SC5314 gDNA with phusion polymerase and cloned into CIp10 at *Mlu*I and *Sal*I sites. Resulting plasmids were linearised with *Nco*I and used to complement the respective homozygous deletion mutants. For creation of the *P*_*ZRT1*_ and *P*_*ZRT2*_ GFP reporters, the upstream intergenic regions of *ZRT1* and *ZRT2* were amplified with phusion polymerase from SC5314 gDNA, cloned into CIp10-GFP [29] at *Xho*I and *Mlu*I sites and verified by sequencing. Resulting plasmids were linearised with *Nco*I and transformed into CAI4 for integration at the *RPS1* locus. In order to localise Zrc1, the protein was tagged at the C-terminus which is predicted to face the cytoplasm (Octopus [56], Phobius [57], and TMHMM [58]), with a Venus yellow fluorescent protein. The Venus sequence was codon optimised for expression in *C*. *albicans* and synthesised (GeneArt), flanked by Pfl23II (5’) and BamHI (3’). The gene was subcloned into pFA-HIS1 at these sites generating pFA-HIS1-Venus. Both Venus and the *HIS1* cassette were amplified with primers ZRC1Ven-FG and ZRC1Ven-RG. These primers include 30 and 29 base pairs sequence homology to the template plasmid for amplification at the 3’, and 100 and 99 bp homology to the *ZRC1* locus, to replace the *ZRC1* stop codon with Venus. The forward primer additionally contained ggtggtggt between locus- and plasmid- specific regions to introduce a 3 × glycine linker between Zrc1 and Venus. The amplified construct was used to replace the remaining stop codon in the *zrc1*Δ/*ZRC1* heterozygote which was then *URA3*-complemented with CIp10 as above. Resulting *zrc1*/*ZRC1*-VENUS strains were successfully cultured in the presence of 250 μM ZnSO_4_ to ensure functionality of the tagged protein.

### Strains and growth conditions

Strains were maintained on YPD agar [1% yeast extract, 2% myco-peptone, 2% D-glucose, 2% agar]. Liquid overnight cultures were grown in YPD or SD medium in a shaking incubator at 30°C and 200 rpm. Transformants were selected on SD agar supplemented with arginine, histidine and/or uridine (each 20 μg ml^-1^) as required. For isolation of the *zrt2*Δ deletion mutant, selection plates were additionally supplemented with 1 mM ZnSO_4_.

*Escherichia coli* was grown on LB agar [1% bacto-tryptone, 0.5% yeast extract, 1% NaCl, 2% agar] and overnight *E*. *coli* cultures were cultivated in a shaking incubator at 37°C and 200 rpm. For selection purposes 50 μg/ml ampicillin were added to solid or liquid LB medium.

### Zinc limited media

To elicit severe zinc restriction, cells were precultured in YPD, washed three time in ultra-pure water and inoculated at OD_600_ (0.05) in 4 ml LZM (limited zinc medium with the components listed in **S1 Table**) in plastic Universal flasks and incubated at 30°C, 200 rpm for three days. For growth experiments in 96 well plates, cells were inoculated to OD_600_ (0.001) and incubated for seven days. For pH-defined LZM, NaOH was added to alkalinise the medium as required and then the media was buffered with 50 mM Na-tartrate (pH4.5) MES (pH 5–6.5) or HEPES (pH 7–8). To determine *ZRT1* and *ZRT2* promoter activity, CAI4+CIp10, *P*_*ZRT1*_-GFP and *P*_*ZRT2*_-GFP strains were cultured overnight in YPD, washed three times with ultra-pure water and inoculated to OD_600_ (1) into pH-buffered LZM in black walled, clear-bottomed 96 well plates and incubated for 16 h. Fluorescence was measured at 485/520 nm and background (CAI4+CIp10) fluorescence subtracted.

To determine metal toxicity, cells from an SD overnight culture were inoculated into SD medium containing indicated metals (starting OD_600_ 0.05) and OD_600_ determined following 24 h incubation at 30°C.

To determine fungal killing, cells were pre-grown in YPD for 24 h, washed twice in 1mM EDTA, twice in ddH20, then inoculated into fresh SD0 medium to a final OD600 = 0.5 for 24 h. After incubation, cells were adjusted to 10^5^ cells/mL in SD0 + 1M ZnSO4 or, as a control, ddH20 for 3 h. Following incubation, cells were washed twice in ddH20, counted and then diluted to 1000 cells/mL in ddH20. Subsequently, 100μl of cell suspension (100 cells) was spread on YPD plates and incubated at 30˚C. Following incubation, CFUs were counted and compared to determine % survival.

### Zinc uptake assays

#### Yeast

Cells were pre-grown in YPD for 24 h, washed twice in 1mM EDTA, twice in ddH20, then inoculated into fresh SD0 medium to a final OD600 = 0.5 for 24 h. After incubation, cells were adjusted to OD600 = 5 in SD0 medium and pulsed with 25μM ZnSO4 at 30˚C with shaking. At indicated time points, 50μl of the supernatant was collected and quantified for zinc using Abcam zinc assay kit.

#### Hyphae

Cells were pre-grown in YPD for 24 h, washed twice in 1mM EDTA, twice in ddH20, then inoculated into fresh SD25 medium to a final OD600 = 0.5 for 24 h. After incubation, cells were washed twice in 1mM EDTA, twice in ddH20, adjusted to 10^6^ cells/mL in RPMI-0 (RPMI + 1mM EDTA, FeCl [6.17 μM], MnSO4 [13.24 μM] and CuSO4 [0.3 μM]), cultured in 12 well tissue culture plates and incubated at 37˚C and 5% CO2 for 24 h. Cells were then washed thrice in PBS and pulsed with RPMI (which was found to contain 3.61 μM Zn^++^) + 25μM ZnSO4 (28.61μM Zn^++^ total) and incubated at 37˚C and 5% CO2. At indicated time points, 50μl of the supernatant was collected and quantified for zinc using Abcam zinc assay kit.

### Intracellular zinc visualisation

To assess zincosomal zinc compartmentalisation, cells were pregrown in YPD, 30°C, 200 rpm for one day, washed three times with distilled water and inoculated into minimal medium without added zinc “SD0” (2% glucose, 0.5% NH_4_SO_4_, 1X YNB without zinc [Formedium]). Whilst this medium does not contain added zinc, it also lacks a chelator, and thus represents moderate zinc depletion.

For microscopy and flow cytometry experiments, cell were inoculated to OD_600_ = 0.05. For the mutant screen, cells were inoculated to OD_600_ = 4. This was to ensure that all strains were at a similar phase of growth, because the *zrt2*Δ mutant grows poorly in the absence of exogenous zinc.

These prestarved cells were then exposed to 25 μM ZnSO_4_ for various times. Pre-pulsed and zinc-pulsed cells were fixed in Histofix, washed in PBS and stained with 25 μM zinquin ethyl ester (Sigma) for 40 minutes. Cells were again washed with PBS and analysed.

For microscopy, cells were additionally stained with Concanavalin A Alexafluor 647 to visualise the cell surface and analysed using DeltaVision microscope using appropriate filters (DAPI and RhTRITC). Original microscopy DV files are in S10 Fig. For the mutant screen, stained and unstained cells were added to the wells of a black-walled clear-bottomed 96 well plate and fluorescence measured at 355/475 nm using a FluoStar plate reader. Measurements were normalised by subtracting the background fluorescence of unstained cells from the stained samples. For flow cytometry, approximately 10^5^ cells were measured using a BD LSRFortessa.

To localise zincosomes and the fungal vacuole, cells from an overnight YPD culture were washed with 1 mM EDTA and then ddH_2_O, incubated in SD0 for 2–3 h. Cells were then incubated with 40 μM FM4-64 and 250 μM ZnSO_4_ for 45 minutes, washed with EDTA then PBS, incubated zinquin for 45 minutes, washed and visualised using a DeltaVision fluorescent microscope.

To visualise vacuolar zinc, cells were pre-grown in YPD for 24 h, washed twice in PBS, and then stained with ZinPyr-1 (10 μM, 1 h, 37°C, 200 rpm, washed twice in PBS and incubated for a further 1 h). Following ZinPyr-1 staining, cells were stained with 40μM FM4-64 in YPD + 1mM ZnSO_4_ for 40 min at 30˚C with shaking in the dark. Following this, cells were washed twice in YPD + 1mM ZnSO4 and subsequently inoculated into YPD + 1mM ZnSO_4_ for 90 min without dye at 30˚C with shaking in the dark. Cells were then imaged using confocal microscopy.

To determine vacuolar import kinetics, wild type *zrc1*Δ cells were pre-grown in YPD for 24 h, washed twice in 1mM EDTA, twice in ddH20, and then inoculated into fresh SD0 medium to a final OD600 = 0.5 for 24 h. After incubation, cells were stained with 10μM ZinPyr-1 in PBS for 1 h at 37˚C with shaking in the dark. Cells were then washed twice in PBS and incubated for a further 1 h at 37˚C with shaking in the dark. Following incubation, cells were pulsed with 25μM ZnSO_4_ in SD0 medium and incubated at 30˚C with shaking in the dark. At indicated time points, 100μl of sample was collected and transferred to a black-bottomed 96 well plate and quantified for ZinPyr-1 fluorescence using a fluorescent microplate reader.

### Galleria infection model

Cells were pre-grown in YPD for 24 h, washed twice in 1mM EDTA, twice in ddH20, then inoculated into fresh SD25 medium to a final OD600 = 0.5 for 24 h. After incubation, cells were washed twice in 1mM EDTA, twice in PBS, adjusted to 5 x 10^6^ cells/mL in PBS, and then 20 μl (1 x 10^5^ cells/mL) injected into the abdominal pro-leg of larvae. Survival of the larvae was monitored on a 12 h basis post-infection.

### Ethics statement

Mice were kept in the animal facility Umeå Centre for Compartive Biology (UCCB). All animal experiments in this study were carried out in strict accordance with the recommendations in the guide for the care and use of laboratory animals conformed to Swedish animal protection laws and applicable guidelines (djurskyddslagen 1988:534; djurskyddsförordningen 1988:539; djurskyddsmyndigheten DFS 2004:4) and with the Swedish animal protection law in a protocol approved by the local Ethical Committee (Umeå djurförsöksetiska nämnd) permit number A79-14.

### Animal experiments

For analysis of in vivo fitness and virulence, C57BL/6 wild-type mice and S100A9-/- mice from the same background were infected intravenously with 5 x 10^5^ CFUs per animal from logarithmically growing *C*. *albicans* cultures. Male and female mice were included in equal numbers for all infections, the average age of the mice was 12–16 weeks.

Mice were sacrificed by cervical dislocation after one or three days of infection. Kidneys and liver were harvested, homogenised and resulting cell suspensions were plated on YPD plates to determine fungal burden.

Neutrophils were isolated as described before [59]. Briefly, C57BL/6 mice were sacrificed by cervical dislocation and femurs and tibia of both hind limbs were dissected. Bone marrow was flushed out with RPMI1640 w/o PR supplemented with 100 μg/ml Carbenicillin and 50 μg/ml Kanamycin (Duchefa, both). After red blood cell lysis, neutrophils were purified using a discontinuous Percoll gradient of 52%, 69% and 78% PBS-buffered Percoll (GE Healtcare). Collected neutrophils from the 69%/78% interface were washed, resuspended in HBSS^-^ and kept on ice. Prior to use, neutrophils were counted using a Vi-CELL cell counter (Beckman Coulter) and diluted to desired concentration in RPMI1640 w/o PR with antibiotics. All following assays were performed in this medium, if not stated otherwise.

Inhibitory capacity of mouse NETs was quantified as explained earlier [60]. 5 x 10^5^ mouse neutrophils were seeded into a 24-well plate. NET formation was induced by 100 nM phorbol myristate acetate in the presence of 1% (V/V) DNase-free mouse serum. Incubation occurred for 20–22 h at 37°C with 5% CO_2_; NET induction was verified microscopically. NET supernatants were gently removed and 500 μl RPMI w/o PR were added containing 5 x 10^4^ *Candida* cells to reach a multiplicity of infection (MOI) of 0.1. Incubation occurred for 20–22 h at 37°C with 5% CO_2._ Fungal viability was assessed by metabolic activity [61]. Briefly, 0.33 mg/ml XTT (2,3-bis (2-methoxy-4-nitro-5-sulfophenyl)-5-[(phenylamino) carbonyl]-2H-tetrazolium hydroxide; Invitrogen) and 27 μg/ml Co-enzyme Q_0_ (Sigma-Aldrich) were added to each well. After an incubation of 15 min at 37°C, the 450 nm absorbance of the supernatants was measured using a Fluostar Omega plate spectrometer (BMG Labtech).

### Statistical analyses

Kidney fungal burden was analysed in IBM SPSS Statistics 24. Normality and homogeneity of variance were first tested, and ANOVA and Kruskal-Wallis tests performed as appropriate for each data set. For growth assays and expression analysis, data were analysed using GraphPad Prism and either Student’s t-test or ANOVA performed as appropriate. For phylogenetic analyses, amino acid sequences were acquired from FungiDB [23] or from the *Candida* Genome Database [24]. To construct phylogenetic trees Phylogeny.fr One Click was used [39,62]: Alignments were performed using MUSCLE, maximum likelihood calculated using PhyML and tree rendering using TreeDyn.

## Supporting information

S1 Table Strains used in this study. All homozygous mutant strains created in the BWP17 (ura3::λimm434/ura3:: λimm434 his1::hisG/his1::hisG arg4::hisG/arg4::hisG) background. GFP reporters created in the CAI4 (*ura3*::imm434/*ura3*::imm434 *iro1/iro1*::imm434) background. **Primers used in this study**. FG and RG were used for deletion construct generation, pFA plasmid annealing site in lowercase; F1, R1 and Int for genotyping; RecF and RecR for revertant construction, restriction sites underlined. **LZM medium composition**. EDTA (1) and sodium citrate (7) stocks were first adjusted to pH 8 and pH 4.2 respectively. Prepared medium was supplemented with FeCl (6.17 μM), MnSO_4_ (13.24 μM) and CuSO_4_ (0.3 μM).
(DOCX)

S1 Fig Morphogenesis analysis. Indicated strains were inoculated into cell culture plates containing liquid 10% foetal calf serum (**a**), RPMI (**b**), or Spider (**c**) media, incubated at 37°C and imaged at indicated times. Alternatively, individual cells were spread onto 2% agar plates containing 10% foetal calf serum (**d**) or 10% RPMI medium (**e**), incubated at 37°C and resultant colonies imaged at day 6. All experiment performed at least twice.
(TIF)

S2 Fig Biofilm formation. Biofilms formed in RPMI (**a**, **b**), SD (**c**) or Spider (**d**) media and metabolic activity measured at 1.5 and 24 h (**a**, **c**) or biomass determined at 72 h. Experiment performed twice in triplicate.
(TIF)

S3 Fig Zrt2-dependence is bypassed at pH 7 and above. Strains from a YPD overnight culture were washed, inoculated into LZM at an OD_600_ of 0.005 and incubated at 30°C for seven days. (A) Growth recovery of *zrt2*Δ occurs at pH 7.0 and above. (B) Growth of all strains in LZM is recovered by addition of zinc (500 μM). Experiments were performed three times. * indicates statistical difference compared to wild type; # indicates statistical difference compared to mutant (P < 0.05, Student’s t-test).
(TIF)

S4 Fig Relationship between environmental pH and zinc import copy number. (a) Map of soil acidity in the contiguous USA from the BONAP website (http://www.bonap.org/), reproduced with permission from Greg Schmidt, 2008, and includes data from the USDA Natural Resource Conservation Service. Pink colouring shows areas with high percentages (50–100%) of acidic soil (pH <6). Endemicity data for *C*. *immitis* (blue) and *H*. *capsulatum* are superimposed. Panel (a) is inspired from our previous analysis in [30]. (b) Phylogenetic tree of predicted plasma membrane zinc transporters in *C*. *albicans*, *C*. parapsilosis, *H*. *capsulatum*, *C*. *neoformans* and *M*. *globosa*, note expansion of Zrt2 orthologues in *C*. *parapsilosis*.
(PDF)

S5 Fig Phylogenetic relationship of zinc transporters in human fungal pathogens. All Zip-type proteins (PF02535) from *S*. *cerevisiae*, *C*. *albicans*, *A*. *fumigatus*, *C*. *neoformans* and *C*. *gattii*. Red circle denotes demonstrated role in pathogenicity in relevant invasive fungal infection model; blue asterisks denote no/minor role in virulence; yellow diamonds denote redundancy.
(PDF)

S6 Fig Zrt2 protects against calprotectin-dependent inhibition of fungal growth during *C*. *albicans*-neutrophil extracellular trap interaction. Indicated strains were incubated with wild type or S100A9-/- -derived NETs or in medium only. Following ~21 hours incubation, metabolic activity was determined by XTT assay. Activity in the presence of both NET groups was determined compared to control conditions in the absence of NETs. Experiment was performed three time. Shown are the actual measurements used to generate the relative activity presented in **Fig 5**.
(TIF)

S7 Fig Effect of zinc and manganese, copper or iron on the growth of wild-type *C*. *albicans* and ZnT deletion mutants. Optical densities of SD overnight cultures were adjusted to 0.05 then incubated for 24 hrs in SD media containing indicated metal concentrations. Data are the mean of two independent experiments, performed in duplicate. Standard deviation (S.D) values are shown in the right hand column.
(PDF)

S8 Fig Zrc1 is required for virulence in a *Galleria* infection model. *Galleria* larvae (10 per group) were infected with 10^5^ *C*. *albicans* cells and monitored every 12 h. Note that whilst wild type result in high mortality, *zrc1*Δ-infected larvae were not killed. Experiment performed twice—here, and in Fig 13.
(TIF)

S9 Fig Zrc1 is dispensable for mouse kidney colonisation. Kidney fungal burden from the mouse infection experiment reported in Fig 12. No significant differences between strains.
(TIF)

S10 Fig Fluorescent microscopy files. Original DV files used to generate Figs 7, 9 and 12.
(ZIP)
