# Supplementary material for: Biphasic zinc compartmentalisation in a human fungal pathogen
Source: PLoS Pathog. 2018 May 4;14(5):e1007013. doi: 10.1371/journal.ppat.1007013 (PMC5955600; doi:10.1371/journal.ppat.1007013)
Supplement: S1 Table — All homozygous mutant strains created in the BWP17 (ura3::λimm434/ura3:: λimm434 his1::hisG/his1::hisG arg4::hisG/arg4::hisG) background. GFP reporters created in the CAI4 (ura3::imm434/ura3::imm434 iro1/iro1::imm434) background. Primers used in this study. FG and RG were used for deletion construct generation, pFA plasmid annealing site in lowercase; F1, R1 and Int for genotyping; RecF and RecR for revertant construction, restriction sites underlined. LZM medium composition. EDTA (1) and sodium citrate (7) stocks were first adjusted to pH 8 and pH 4.2 respectively. Prepared medium was supplemented with FeCl (6.17 μM), MnSO4 (13.24 μM) and CuSO4 (0.3 μM). (DOCX) [file ppat.1007013.s001.docx]

**Strains used in this study**. All homozygous mutant strains created in the BWP17 (ura3::λimm434/ura3:: λimm434 his1::hisG/his1::hisG arg4::hisG/arg4::hisG ) background. GFP reporters created in the CAI4 (*ura3*::imm434/*ura3*::imm434 *iro1/iro1*::imm434) background.

| **Strain name** | **Genotype** | **Reference** | **Unique identifier (Wilson lab)** |
| --- | --- | --- | --- |
| BWP17 | ura3::λimm434/ura3:: λimm434 his1::hisG/his1::hisG arg4::hisG/arg4::hisG | Wilson et al. (1999) | D1 |
| BWP17+CIp30 isogenic “wild type” | ura3::λimm434/ura3:: λimm434 his1::hisG/his1::hisG arg4::hisG/arg4::hisG  +CIp30 | Mayer et al. (2012) | D2 |
| *zrt1*Δ | *zrt1*::*HIS1*/*zrt1*::*ARG4* +CIp10 | Citiulo et al., (2012) | D3 |
| *zrt1*Δ+*ZRT1* | *zrt1*::*HIS1*/*zrt1*::*ARG4* +Cip10-*ZRT1* | Citiulo et al., (2012) | D4 |
| *zrt2*Δ | *zrt2*::*HIS1*/*zrt2*::*ARG4* +CIp10 | This study | D9 |
| *zrt2*Δ+*ZRT2* | *zrt2*::*HIS1*/*zrt2*::*ARG4* +CIp10-*ZRT2* | This study | D10 |
| *zrt1*∆*zrt2*∆ | *zrt1*::*HIS1*/*zrt1*::*ARG4 zrt2*::*FRT*/*zrt2*::*FRT*+CIp10 | This study | D87 |
| *zrt1*∆*zrt2*∆+*ZRT1ZRT2* | *zrt1*::*HIS1*/*zrt1*::*ARG4 zrt2*::*FRT*/*zrt2*::*FRT*+CIp10-*ZRT1*-*ZRT2* | This study | D85 |
| *P_ZRT2_*-GFP | *ura3*::imm434/*ura3*::imm434 *iro1/iro1*::imm434 +CIp10-*P_ZRT2_*-GFP | This study | D26 |
| *P_ZRT1_*-GFP | *ura3*::imm434/*ura3*::imm434 *iro1/iro1*::imm434 +CIp10-*P_ZRT1_*-GFP | This study | D30 |
| CAI4+CIp10 | *ura3*::imm434/*ura3*::imm434 *iro1/iro1*::imm434 +CIp10 | Fradin et al., (2005) | D101 |
| *zrt2*Δ+*ZRT2* | *zrt2*::*HIS1*/*zrt2*::*ARG4* +CIp10-*ZRT2* | This study | D10 |
| *zrc1*Δ/*ZRC1* | orf19.1536::*HIS1*/orf19.1536 | This study | D123 |
| *zrc1*Δ/*ZRC1*-VENUS | orf19.1536::*HIS1*/orf19.1536-VENUS-*ARG4* + CIp10 | This study | D157 |
| *zrc1*Δ | orf19.1536::*HIS1*/orf19.1536+CIp10 | This study | D11 |
| *zrc1*Δ+*ZRC1* | orf19.1536::*HIS1*/orf19.1536+CIp10-*ZRC1* | This study | D12 |
| orf19.3874Δ | orf19.3874::*HIS1*/orf19.3874::*ARG4*+CIp10 | This study | D97 |
| orf19.3769Δ | orf19.3769::*HIS1*/orf19.3769::*ARG4*+CIp10 | This study | D116 |
| orf19.3132Δ | orf19.3132::*HIS1*/orf19.3132::*ARG4*+CIp10 | This study | D62 |
| orf19.52Δ | orf19.52::*HIS1*/orf19.52::*ARG4*+CIp10 | This study | D99 |

**Primers used in this study**. FG and RG were used for deletion construct generation, pFA plasmid annealing site in lowercase; F1, R1 and Int for genotyping; RecF and RecR for revertant construction, restriction sites underlined.

|  |  |  |
| --- | --- | --- |
| ZRT2-FG | GTCAAATTGATTACTTTTATTGAGCTCTCCTTCATTTTGTTCTCATCATAGTTGGCTTTCATTATCAATTCCCAGCATAAAATTAGTTAACACTGACCCTGACT**gaagcttcgtacgctgcaggtc** | This study |
| ZRT2-RG | ATATCAAAATGAATATAAGAAAAGAAAAATATTAATCTATAAATTCAAACTTGAATGAAATTAAAACGCATAAAAAGATATAATGAAAACACAAGTGTCCGTGC**tctgatatcatcgatgaattcgag** | This study |
| ZRT2-F1 | GTCGGATTGGGTTACATTCAGATTG | This study |
| ZRT2-R1 | CTTCTGCTCGCTTCAAAGTTGAC | This study |
| ZRT2-IntF1 | CCAAGATCCAGACGTGATGGGGAC | This study |
| ZRT2-RecF1 | GGTCGACGAGTGTTTGCTGCAATC | This study |
| ZRT2-RecR1 | AGTACGCGTATCATCTAGTACTGTGC | This study |
| ProZRT1-F1 | ATCTCGAGAGAAAGTTTGTGATTATAGATGC | This study |
| ProZRT1-R1 | TGAACGCGTTAATTTTTAGAGAGCTACCAC | This study |
| Pro-ZRT2-F1 | ATACTCGAGCGAGTGTTTGCTGCAATC | This study |
| Pro-ZRT2-R1 | AGTACGCGTCAGTGTTAACTAATTCTATGC | This study |
| ZRC1-FG | ATTCTTATTATTATCACGATCAATTAATTTAAAATTCCGATTTTTTTTTTTTGGTTTTTTTTGTTTTTTCTTATTTTATTTTATTTTATTTTCCAACAGACAAC**gaagcttcgtacgctgcaggtc** | This study |
| ZRC1-RG | ATATTTGCTATAATTACATGAATAATATTCCAATATAAATTATAATTAACATACAAATATATATTCATATATGGTTCAATGTTTAATTCTATTTTTTTTTTTTT**tctgatatcatcgatgaattcgag** | This study |
| ZRC1-F1 | TTGTGTGAATCGGCCAGCCGGGAG | This study |
| ZRC1-R1 | CCTCCAGTTAACAAGGCATAAGATGG | This study |
| ZRC1-IntR1 | CCGTATCAAGAATTAATAACGCAAC | This study |
| ZRC1-RecF1 | GTAACGCGTGGGAGTAAAATTGTTG | This study |
| ZRC1-RecR1 | CCGTCGACTGAAGTCAATCCTCCTCC | This study |
| orf19.3874-FG | GGTTTGTCAAATTTTTTAATACAAATTGCCACTTGTTGTTATCATTTGATTATATAAGATAAGATCTACTGTACTTTCTTTTATTCCTTAGTTATCAACCAATAgaagcttcgtacgctgcaggtc | This study |
| orf19.3874-RG | GATATAATTGTCACTTACAAAAATAATCTAGAAACTTGTTTAAAAGAAAATTTTATATAATTAGGATTATTAATTCTATTTAATTGGTTTATTTATTTATATATtctgatatcatcgatgaattcgag | This study |
| orf19.3874-F1 | CGAAATGGACGGCGTATATTCC | This study |
| orf19.3874-R2 | GGTCGGATGGTTAGTGAAATTGG | This study |
| orf19.3769-FG | TTGAAAAAAGGGGAACAAATAAAAACCCAAAACTGACAGCATTCTTCAAATCACATAATTTGTCTTAATTATTCCTATCACCACATCAGCCCTAATTATATTCAgaagcttcgtacgctgcaggtc | This study |
| orf19.3769-RG | TTACTCGATATGGAAGTTGCGGCTGTCCTGCACCCCAAATGTACTTGGCTCAACTCATATCACTACACAGTTTACTACTAGATTAGGTAAATATATTTATTTTTtctgatatcatcgatgaattcgag | This study |
| orf19.3769-F3 | GCTGCTTTCATCCTGCCAAGG | This study |
| orf19.3769-R1 | CGATAAAGTTGCTGTGTTGAACAGG | This study |
| orf19.3132-FG | GAAAAAAAAGGAAAGCAAGTTATCAACAACACCTCTCAACTTCCTCCCCCTACCCCCCAGATACACTTATAGATAGATTCATACATATTCAGTTAATTTTTAATgaagcttcgtacgctgcaggtc | This study |
| orf19.3132-RG | AGTTTGTAATTAAACGGATTGTTTTTTTTTTTGGCTATATTAAATTAGTTATTAAATAGTAAAAGGGGGATACAAAATGAACCCACTAACCGACAATCCTGTTCtctgatatcatcgatgaattcgag | This study |
| orf19.3132-F1 | CGGAAGAAAGGGACAAAGTGCG | This study |
| orf19.3132-R2 | CCAGGTTTGGTATGGGAGTTTGC | This study |
| orf19.52-FG | GTGCATACAGTACATTAATAATATATGTATTAGTATTAGTATTAACTTTTTAACATTTGGACACTAGAAAAATTTATTTTCCCTCCACTTCCCTTTTCTATATAgaagcttcgtacgctgcaggtc | This study |
| orf19.52-RG | GTCGAGGTTATTGTTAAAACAAGTAGAGGAGTGGAAAACTTTGCATATATATAAGAAGATTTATAAATAAAAAAAAAATAACTTTTCTTTTGGGATACAGTCTAtctgatatcatcgatgaattcgag | This study |
| orf19.52-F1 | GGAAGAAATCGGAACATCACAATTGG | This study |
| orf19.52-R1 | GCAACTAGAGACTTCTGCTTATGG | This study |
| RPF-F1 | gagcagtgtacacacacacatcttg | Wilson et al. (2014) |
| RPF1-R1 | cgccaaagagtttcccctattatc | This study |
| URA3-F2 | ggagttggattagatgataaaggtgatgg | Gola et al. (2003) |
| HIS1-F2 | ggacgaattgaagaaagctggtgcaaccg | Gola et al. (2003) |
| HIS1-R2 | caacgaaatggcctcccctaccacag | Gola et al. (2003) |
| ARG4-F2 | ggatatgttggctactgatttagc | Martin et al., (2007) |
| ARG4-R2 | aatggatcagtggcaccggtg | Gola et al. (2003) |
| FPzrt2_interg_candida | TTGACGATTTTTGGTGACCT | This  study |
| RPzrt2_integr_candida | ACTTCTGCTCGCTTCAAAGTT | This study |
| RPF-4 | CGCCAAAGAGTTTCCCCTAT | This study |
| RafterZRT2incandida | ctttatgcttccggctcgta | This study |
| ZRC1Ven-FG | tgttggtcaagttagagaagttttacatatttatggtattaattcagttactatacaacctgaatttctgaatagtaaaaaagtgtgtcgtctgattttcggtggtggtGTTTCAAAAGGTGAAGAATTATTCACTGGT | This study |
| ZRC1Ven-RG | aacatataatatttgctataattacatgaataatattccaatataaattataattaacatacaaatatatattcatatatggttcaatgtttaattctaTGGATCTGATATCATCGATGAATTCGAGC | This study |

**LZM medium composition**. EDTA (1) and sodium citrate (7) stocks were first adjusted to pH 8 and pH 4.2 respectively. Prepared medium was supplemented with FeCl (6.17 µM), MnSO_4_ (13.24 µM) and CuSO_4_ (0.3 µM).

| **Stock** | **Fold conc.** | **Component** | **Stock conc. (M)** | **Final conc. (M)** | **Vol.** |
| --- | --- | --- | --- | --- | --- |
| 1 | 500 | Na_2_EDTA.2H_2_O | 5.0x10^-1^ | 1.0x10^-3^ | 1ml |
| 2 | 100 | MgSO_4_.7H_2_O  NaCl | 5.0x10^-1^  1.0x10^-1^ | 5.0x10^-3^  1.0x10^-3^ | 5ml |
| 3 | 100 | CaCl_2_.2H_2_O | 1.0x10^-1^ | 1.0x10^-3^ | 5ml |
| 4 | 100 | Uridine  L-Histidine  L-Leucine  L-Lysine | 4.0x10^-2^  5.0x10^-2^  7.6x10^-2^  7.0x10^-2^ | 4.0x10^-4^  5.0x10^-4^  7.6x10^-4^  7.0x10^-4^ | 5ml |
| 5 | 100 | (NH_4_)_2_SO_4_ | 3.8 | 3.8x10^-2^ | 5ml |
| 6 | 100 | KH_2_PO_4_ | 1.0x10^-1^ | 1.0x10^-3^ | 5ml |
| 7 | 50 | Na_3_Citrate.2H_2_O | 1.0 | 2.0x10^-2^ | 10ml |
| 8 | 20 | D-glucose | 2.2x10^-1^ | 1.1x10^-2^ | 25ml |
| 9 | 1000 | d-Biotin  Ca Pantothenate  myo-Inositol  Pyridoxin  Thiamin.HCl | 1.6x10^-5^  1.7x10^-3^  1.0x10^-2^  2.0x10^-3^  1.0x10^-3^ | 1.6x10^-8^  1.7x10^-6^  1.0x10^-5^  2.0x10^-6^  1.0x10^-6^ | 0.5ml |
| 10 | 10000 | H_3_BO_3_  KI  Na_2_MoO_4_.2H_2_O | 1.0x10^-1^  5.0x10^-3^  1.0x10^-2^ | 1.0x10^-5^  5.0x10^-7^  1.0x10^-6^ | 50µl |
