# Supplementary figures and images for: Biphasic zinc compartmentalisation in a human fungal pathogen
Source: PLoS Pathog. 2018 May 4;14(5):e1007013. doi: 10.1371/journal.ppat.1007013 (PMC5955600; doi:10.1371/journal.ppat.1007013)

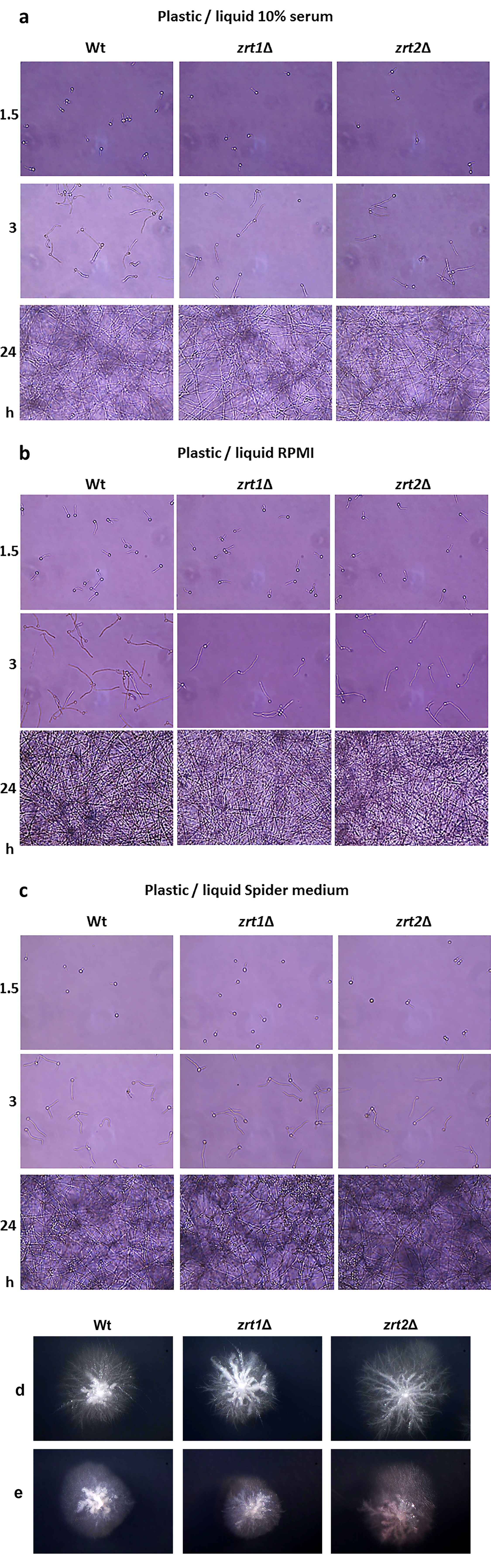

Supplement: S1 Fig — Indicated strains were inoculated into cell culture plates containing liquid 10% foetal calf serum (a), RPMI (b), or Spider (c) media, incubated at 37°C and imaged at indicated times. Alternatively, individual cells were spread onto 2% agar plates containing 10% foetal calf serum (d) or 10% RPMI medium (e), incubated at 37°C and resultant colonies imaged at day 6. All experiment performed at least twice. (TIF) [file ppat.1007013.s002.tif]

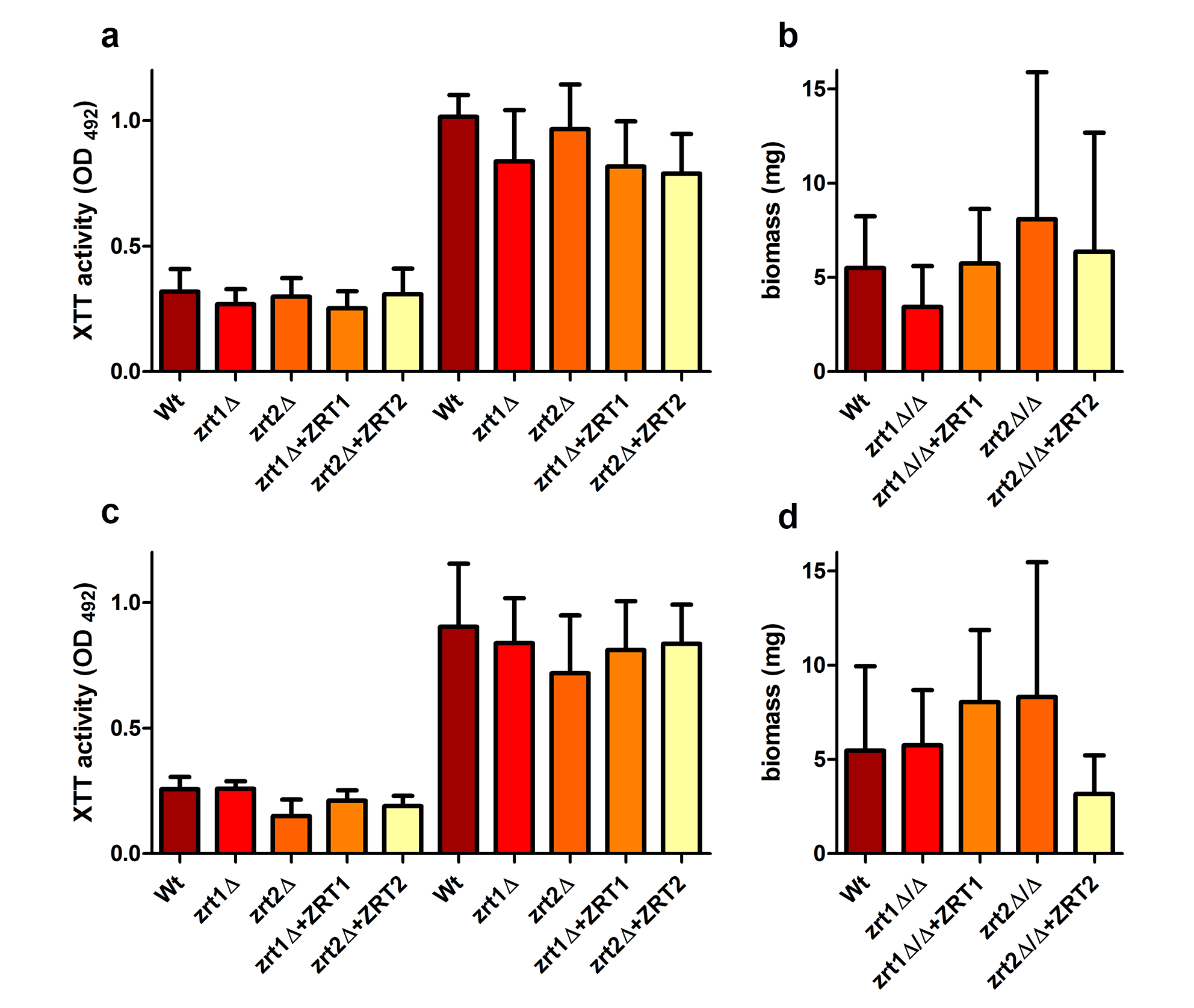

Supplement: S2 Fig — Biofilms formed in RPMI (a, b), SD (c) or Spider (d) media and metabolic activity measured at 1.5 and 24 h (a, c) or biomass determined at 72 h. Experiment performed twice in triplicate. (TIF) [file ppat.1007013.s003.tif]

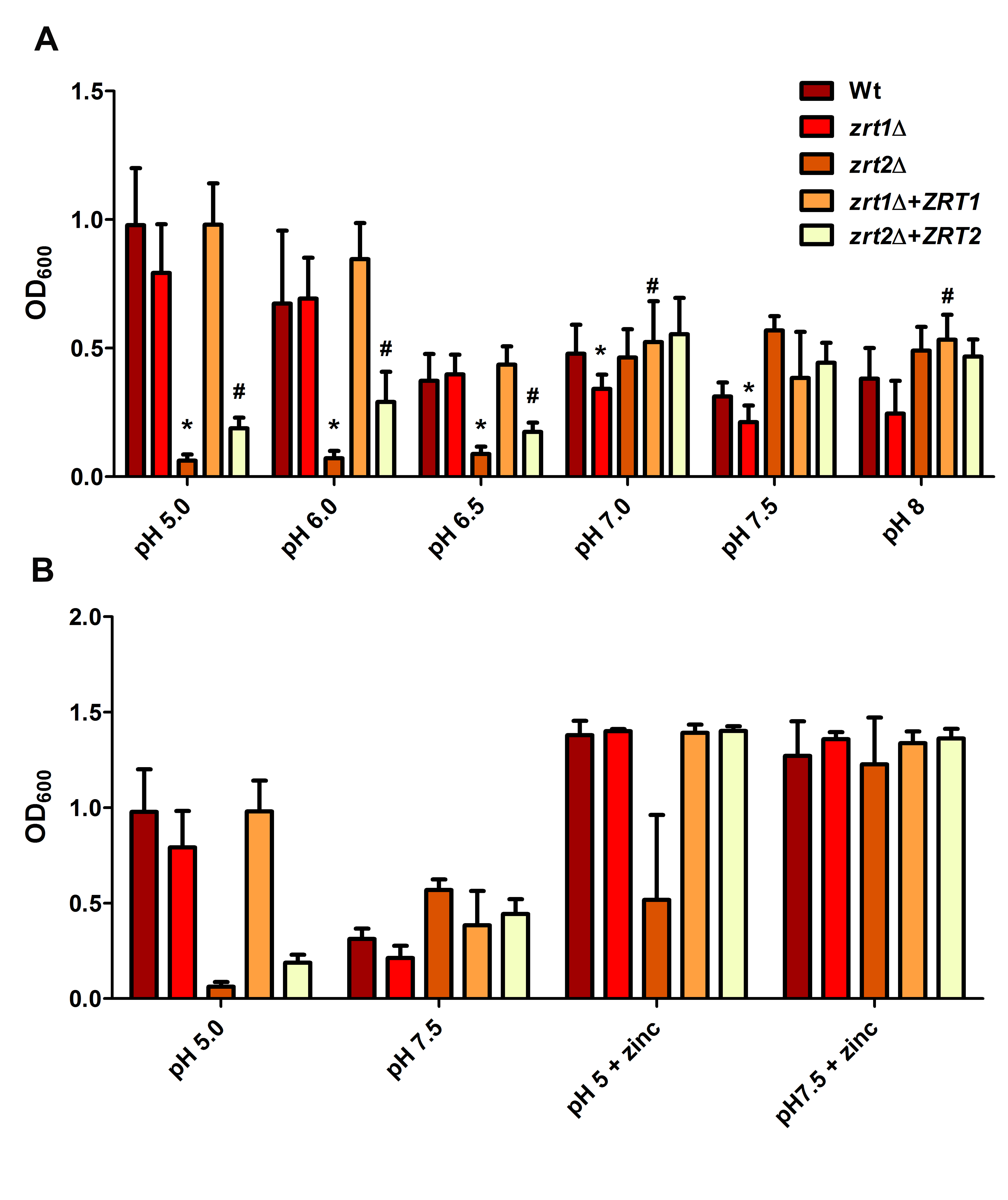

Supplement: S3 Fig — Strains from a YPD overnight culture were washed, inoculated into LZM at an OD600 of 0.005 and incubated at 30°C for seven days. (A) Growth recovery of zrt2Δ occurs at pH 7.0 and above. (B) Growth of all strains in LZM is recovered by addition of zinc (500 μM). Experiments were performed three times. * indicates statistical difference compared to wild type; # indicates statistical difference compared to mutant (P < 0.05, Student’s t-test). (TIF) [file ppat.1007013.s004.tif]

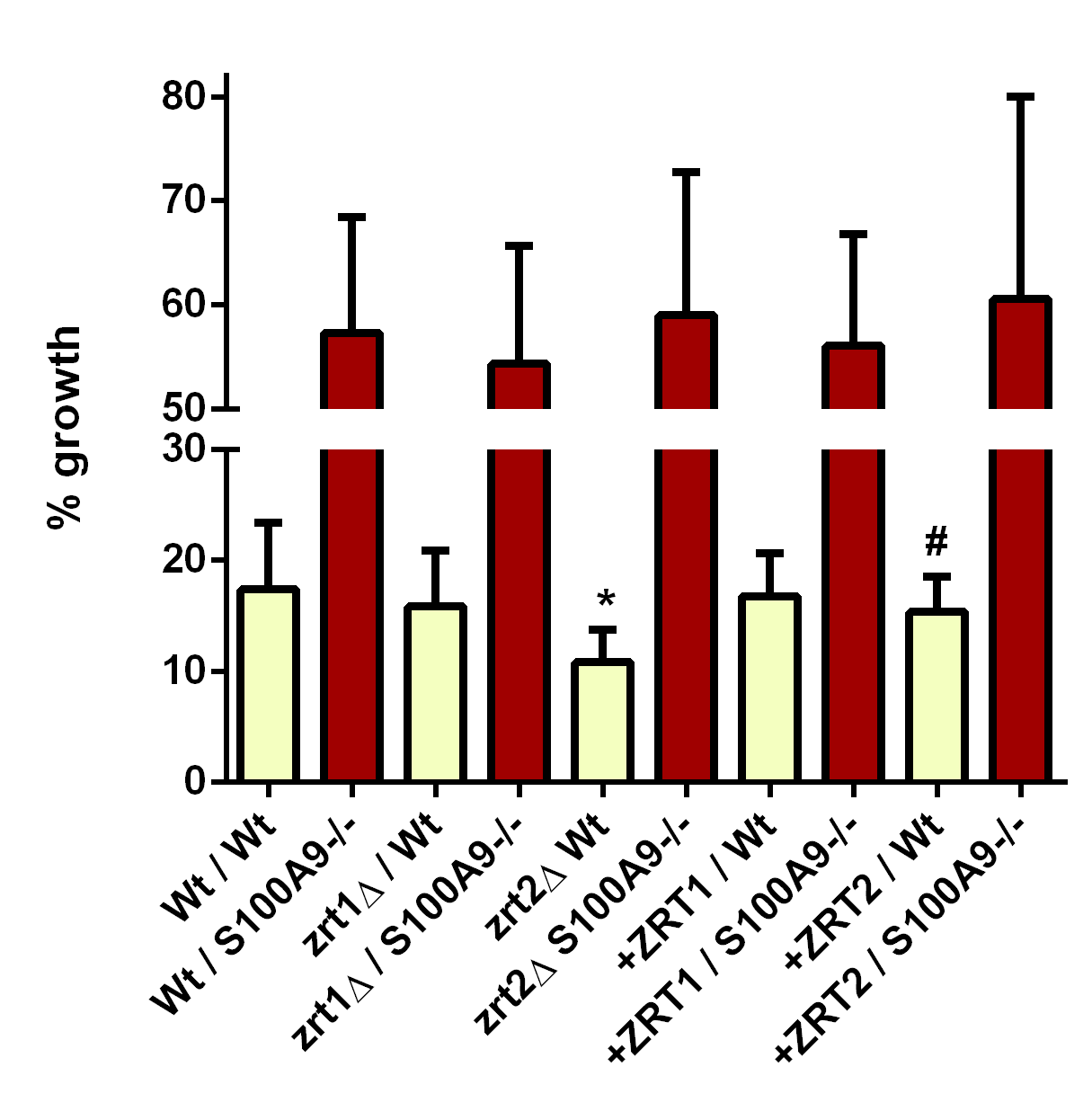

Supplement: S6 Fig — Indicated strains were incubated with wild type or S100A9-/- -derived NETs or in medium only. Following ~21 hours incubation, metabolic activity was determined by XTT assay. Activity in the presence of both NET groups was determined compared to control conditions in the absence of NETs. Experiment was performed three time. Shown are the actual measurements used to generate the relative activity presented in Fig 5. (TIF) [file ppat.1007013.s007.tif]
