# Supplementary material for: Biphasic zinc compartmentalisation in a human fungal pathogen
Source: PLoS Pathog. 2018 May 4;14(5):e1007013. doi: 10.1371/journal.ppat.1007013 (PMC5955600; doi:10.1371/journal.ppat.1007013)
Supplement: S4 Fig — (a) Map of soil acidity in the contiguous USA from the BONAP website (http://www.bonap.org/), reproduced with permission from Greg Schmidt, 2008, and includes data from the USDA Natural Resource Conservation Service. Pink colouring shows areas with high percentages (50–100%) of acidic soil (pH <6). Endemicity data for C. immitis (blue) and H. capsulatum are superimposed. Panel (a) is inspired from our previous analysis in [30]. (b) Phylogenetic tree of predicted plasma membrane zinc transporters in C. albicans, C. parapsilosis, H. capsulatum, C. neoformans and M. globosa, note expansion of Zrt2 orthologues in C. parapsilosis. (PDF) [file ppat.1007013.s005.pdf]

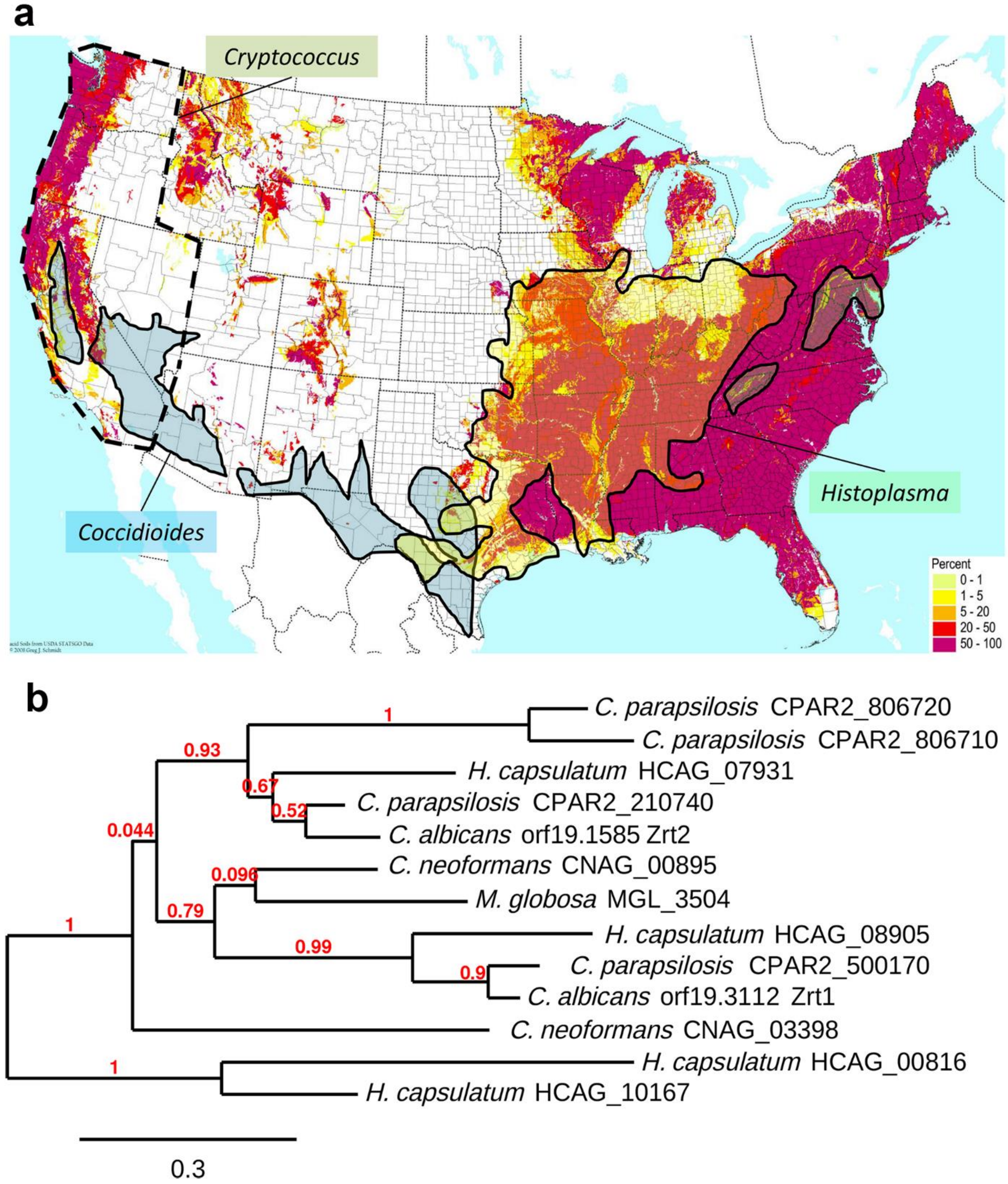

Potential impact of ecological niche pH on zinc assimilation genes

Our observations that pH-dependence of zinc transporter expression/function is conserved in the relatively distantly related species, *C. albicans* and *A. fumigatus*, prompted us to consider this relationship further. Specifically, we hypothesised that ecological pH adaptation may have shaped the evolution of zinc assimilation in the fungal kingdom.

We selected several species known to inhabit acidic environments (see below) and compared their zinc import genes. These were: *Cryptococcus neoformans*, *Histoplasma capsulatum*, *Candida parapsilosis* and *Malassezia globosa*. These species represent members of both the Ascomycota and the Basidiomycota, which are the two major phyla of the fungal kingdom and diverged at least 452 million years ago [1].

The basidiomycete, *Cryptococcus neoformans* is often isolated from bird-guano-enriched soil and has been associated with (particularly citric) fruits [2]. The related species, *C. gattii* is endemic to the North-western seaboard of the United States (**a**). Like *C. neoformans*, the ascomycete fungus *Histoplasma capsulatum* is also endemic to areas of acidic soil (**a**). The ascomycete and basidiomycete yeasts, *C. parapsilosis* and *M. globosa*, respectively, colonise human skin, which is mildly acidic. *C. neoformans* has maintained single copies of two ancestral zinc importer genes (**b**; see also discussed below, **Figure S5**). The second basidiomycete, *M. globosa*, encodes only a single zinc importer, which is orthologous to the acid-adapted Zrt2 in *C. albicans*. This suggests that this skin-colonising species has lost its Zrt1 orthologue, which is neutral-alkaline adapted in the ascomycetes *C. albicans* and *A. fumigatus*. At this stage it is not clear whether pH-regulation of zinc transporters occurs in the Basidiomycota - such regulation has not been reported in *C. neoformans* [3] and the transporter orthologues in the related species, *C. gattii*, do not appear to be pH-dependent [4]. The environmental ascomycete *H. capsulatum* has maintained both Zrt1 and Zrt2 orthologues, and undergone expansion of an as-yet uncharacterised transporter sub-class. Finally, *C. parapsilosis* has also maintained both Zrt1 and Zrt2 orthologues. Interestingly, *C. parapsilosis* has undergone expansion of *ZRT2* (**b**). It is possible that triplication of a gene essential for growth in acidic environments (**Figure 1**) increases *C. parapsilosis* fitness on skin and we are currently investigating this hypothesis in greater detail.

As well as zinc transporters, we and others have found that the secreted zinc-binding "zincophore" protein, encoded by *PRA1* in *C. albicans* and *aspF2* in *A. fumigatus*, is upregulated and functionally active at neutral-alkaline pH, but transcriptionally inactive in acidic environments [5-7]. Indeed a recent bioinorganic chemistry study has demonstrated [neutral] pH specificity of Pra1-Zn^{++} binding [8]. Strikingly, all four acid-adapted species have lost the zincophore gene.

These gene loss events appear to be independent, as the basidiomycete *Ustilago maydis*, which is more closely related to *Malassezia* than to *Cryptococcus* [9], maintains a *PRA1* orthologue [5]. Similarly, several *Candida* species, including both close, and more distant relations of *C. parapsilosis*, have maintained a *PRA1* orthologue [5]. Perhaps the most interesting example is that of *H. capsulatum*. This fungus is relatively closely related to another endemic pathogen, *Coccidioides immitis*, a species which has maintained the zincophore gene. Geographical analysis of their regions of endemicity reveal almost exact mapping to areas of respective acidic and alkaline soils in the contiguous USA (**Figure 6**) [10]. As CaPra1 expression can be deleterious in some host niches [11,12], these gene loss events in *C. parapsilosis* and *M. globosa* may have also been driven by immune-selection in these mammalian-adapted fungal species.

In summary, an environmental basidiomycete (*C. neoformans*) and ascomycete (*H. capsulatum*), and skin-associated basidiomycete (*M. globosa*) and ascomycete (*C. parapsilosis*), have independently lost the zincophore gene. Moreover, *C. parapsilosis* has undergone expansion of its *ZRT2* orthologue, a gene essential for growth in acidic media in its relative, *C. albicans* (**Figure 1**). Based on these examples, we propose that adaptation to

environmental pH has conceivably shaped the evolution of zinc uptake gene copy number in extant fungal species. In the future, it will be important to test whether ecological adaptation has selected for particular pathophysiological lifestyles such as macrophage parasitism in *Cryptococcus* and *H. capsulatum* [13].

1. Taylor JW, Berbee ML (2006) Dating divergences in the Fungal Tree of Life: review and new analyses. Mycologia 98: 838-849.
2. Lopez-Martinez R, Castanon-Olivares LR (1995) Isolation of *Cryptococcus neoformans* var. *neoformans* from bird droppings, fruits and vegetables in Mexico City. Mycopathologia 129: 25-28.
3. Do E, Hu G, Caza M, Kronstad JW, Jung WH (2016) The ZIP family zinc transporters support the virulence of *Cryptococcus neoformans*. Med Mycol 54: 605-615.
4. Schneider Rde O, Diehl C, Dos Santos FM, Piffer AC, Garcia AW, Kulmann MI, Schrank A, Kmetzsch L, Vainstein MH, Staats CC (2015) Effects of zinc transporters on *Cryptococcus gattii* virulence. Sci Rep 5: 10104.
5. Citiulo F, Jacobsen ID, Miramon P, Schild L, Brunke S, Zipfel P, Brock M, Hube B, Wilson D (2012) *Candida albicans* scavenges host zinc via Pra1 during endothelial invasion. PLoS Pathog 8: e1002777.
6. Amich J, Vicentefranqueira R, Leal F, Calera JA (2010) *Aspergillus fumigatus* survival in alkaline and extreme zinc-limiting environments relies on the induction of a zinc homeostasis system encoded by the *zrfC* and *aspf2* genes. Eukaryot Cell 9: 424-437.
7. Bensen ES, Martin SJ, Li M, Berman J, Davis DA (2004) Transcriptional profiling in *Candida albicans* reveals new adaptive responses to extracellular pH and functions for Rim101p. Mol Microbiol 54: 1335-1351.
8. Loboda D, Rowinska-Zyrek M (2017) Zinc binding sites in Pra1, a zincophore from *Candida albicans*. Dalton Trans.
9. Matheny PB, Wang Z, Binder M, Curtis JM, Lim YW, Nilsson RH, Hughes KW, Hofstetter V, Ammirati JF, Schoch CL, Langer E, Langer G, McLaughlin DJ, Wilson AW, Froslev T, Ge ZW, Kerrigan RW, Slot JC, Yang ZL, Baroni TJ, Fischer M, Hosaka K, Matsuura K, Seidl MT, Vauras J, Hibbett DS (2007) Contributions of *rpb2* and *tef1* to the phylogeny of mushrooms and allies (Basidiomycota, Fungi). Mol Phylogenet Evol 43: 430-451.
10. Wilson D (2015) An evolutionary perspective on zinc uptake by human fungal pathogens. Metallomics.
11. Soloviev DA, Fonzi WA, Sentandreu R, Pluskota E, Forsyth CB, Yadav S, Plow EF (2007) Identification of pH-regulated antigen 1 released from *Candida albicans* as the major ligand for leukocyte integrin alphaMbeta2. J Immunol 178: 2038-2046.
12. Soloviev DA, Jawhara S, Fonzi WA (2011) Regulation of innate immune response to *Candida albicans* infections by alphaMbeta2-Pra1p interaction. Infect Immun 79: 1546-1558.
13. Seider K, Heyken A, Luttich A, Miramon P, Hube B (2010) Interaction of pathogenic yeasts with phagocytes: survival, persistence and escape. Curr Opin Microbiol 13: 392-400.
