# Supplementary material for: Biphasic zinc compartmentalisation in a human fungal pathogen
Source: PLoS Pathog. 2018 May 4;14(5):e1007013. doi: 10.1371/journal.ppat.1007013 (PMC5955600; doi:10.1371/journal.ppat.1007013)
Supplement: S5 Fig — All Zip-type proteins (PF02535) from S. cerevisiae, C. albicans, A. fumigatus, C. neoformans and C. gattii. Red circle denotes demonstrated role in pathogenicity in relevant invasive fungal infection model; blue asterisks denote no/minor role in virulence; yellow diamonds denote redundancy. (PDF) [file ppat.1007013.s006.pdf]

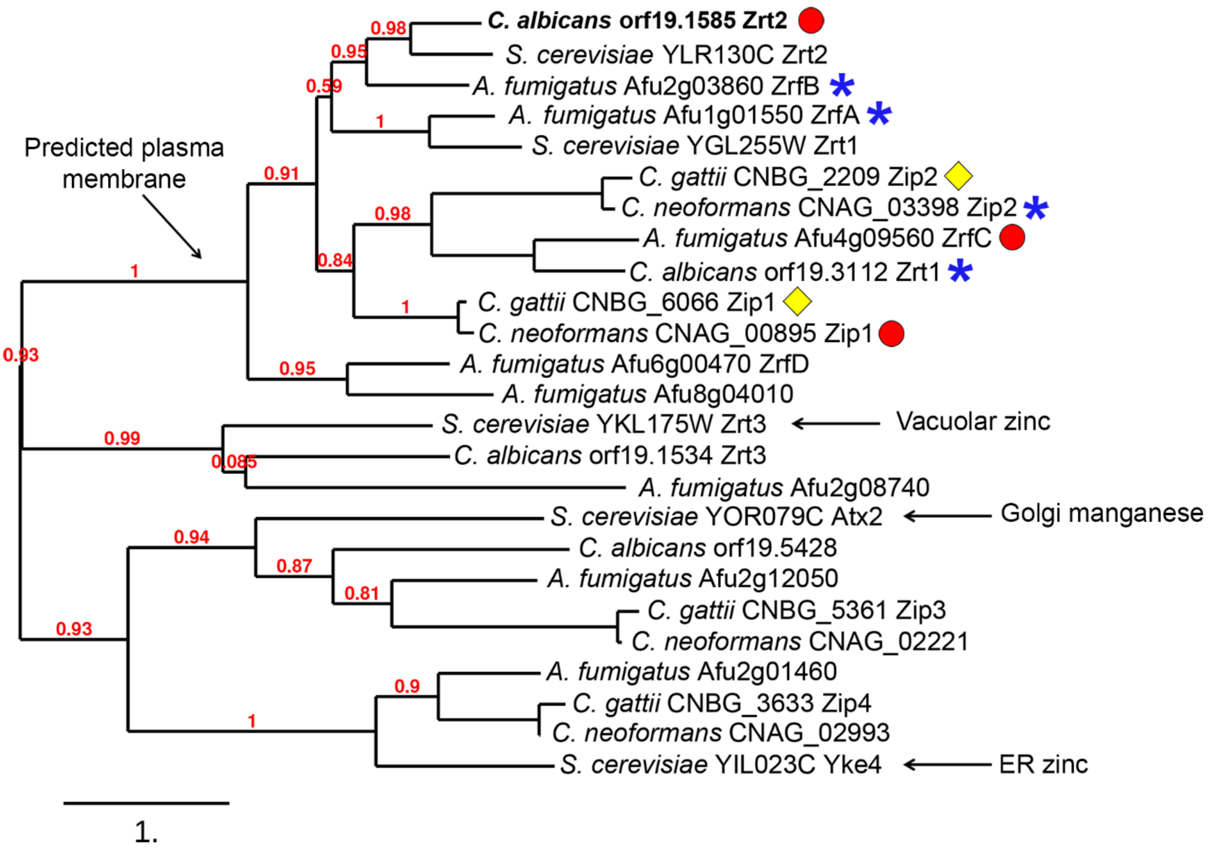

Phylogenetic predictions of pathogenicity

Given the importance of zinc assimilation for fungal pathogenicity, we assessed the conservation of zinc transporters in these human pathogenic species. **Tree** shows the phylogenetic relationship between zinc transporter proteins of *C. albicans*, *A. fumigatus*, *C. neoformans* and *C. gattii* and highlights those transporters with a demonstrated role in pathogenicity.

In *A. fumigatus*, deletion of *zrfC* attenuates virulence and, whilst a *zrfABC*Δ triple mutant was avirulent, genetic complementation of this strain with *zrfC* restored lethality to wild type levels [1]. Although single infection studies with *zrfA*Δ and *zrfB*Δ mutants have not been reported, the wild type virulence of *zrfABC*Δ+*zrfC* suggests that zinc assimilation via ZrfC dominates during invasive pulmonary aspergillosis and that *zrfA* and *zrfB* are dispensable for pathogenicity in these models (blue asterisks).

In *C. neoformans*, deletion of *ZIP1*, but not *ZIP2* attenuated virulence, and a *zip1*Δ/*zip2*Δ double deletion mutant was only moderately more attenuated than *zip1*Δ [2]. In contrast, in the related species, *C. gattii*, only a *zip1*Δ/*zip2*Δ double mutant exhibited attenuated virulence with both *zip1*Δ and *zip2*Δ single mutants being as virulent as the wild type [3].

We have previously shown that *C. albicans* Zrt1, and the co-expressed zincophore Pra1, are involved in zinc assimilation from endothelial monolayers, yet *Ca*Zrt1 is dispensable for kidney colonisation (this study and [4]). In contrast, we now show *Ca*Zrt2 is the dominant zinc importer during kidney colonisation.

Therefore, different species require different transporters or transporter combinations to thrive in their host and cause disease. We postulate that ecological adaptation may have shaped the evolution of the zinc transport arsenal of extant fungal species (**Figure S4** and [5]). These species-specific mechanisms make dissection of the role of zinc assimilation on pathogenic potential a challenging and exciting field of study.

1. Amich J, Vicentefranqueira R, Mellado E, Ruiz-Carmuega A, Leal F, Calera JA (2014) The ZrfC alkaline zinc transporter is required for *Aspergillus fumigatus* virulence and its growth in the presence of the Zn/Mn-chelating protein calprotectin. Cell Microbiol 16: 548-564.
2. Do E, Hu G, Caza M, Kronstad JW, Jung WH (2016) The ZIP family zinc transporters support the virulence of *Cryptococcus neoformans*. Med Mycol 54: 605-615.
3. Schneider Rde O, Diehl C, Dos Santos FM, Piffer AC, Garcia AW, Kulmann MI, Schrank A, Kmetzsch L, Vainstein MH, Staats CC (2015) Effects of zinc transporters on *Cryptococcus gattii* virulence. Sci Rep 5: 10104.
4. Noble SM, French S, Kohn LA, Chen V, Johnson AD (2010) Systematic screens of a *Candida albicans* homozygous deletion library decouple morphogenetic switching and pathogenicity. Nat Genet 42: 590-598.
5. Wilson D (2015) An evolutionary perspective on zinc uptake by human fungal pathogens. Metallomics.
