# Supplementary material for: Biphasic zinc compartmentalisation in a human fungal pathogen
Source: PLoS Pathog. 2018 May 4;14(5):e1007013. doi: 10.1371/journal.ppat.1007013 (PMC5955600; doi:10.1371/journal.ppat.1007013)
Supplement: S7 Fig — Optical densities of SD overnight cultures were adjusted to 0.05 then incubated for 24 hrs in SD media containing indicated metal concentrations. Data are the mean of two independent experiments, performed in duplicate. Standard deviation (S.D) values are shown in the right hand column. (PDF) [file ppat.1007013.s008.pdf]

Manganese concentration (mM)

Zinc concentration (mM)

Wild-type

	0	0.01	0.1	1	10	100
0	1.043	1.028	1.045	1.042	0.1338	0.0942
0.01	1.036	1.037	1.009	1.029	0.1481	0.1004
0.1	0.9708	1.001	0.9738	1.027	0.1656	0.1006
1	0.9928	0.9842	0.9964	1.016	0.1717	0.1006
10	0.934	0.9473	0.969	0.9669	0.1721	0.1018
100	0.8388	0.8642	0.8763	0.8276	0.145	0.1032

S.D

0.02739	0.02673	0.01646	0.0267	0.01885	0.001556
0.0311	0.02871	0.1055	0.02536	0.01357	0.002867
0.06892	0.04129	0.07405	0.04773	0.02426	0.003176
0.0367	0.03104	0.02157	0.03872	0.008375	0.006093
0.0369	0.05646	0.04597	0.04675	0.0201	0.005291
0.05091	0.05501	0.05073	0.1137	0.02892	0.00341

zrc1Δ

	0	0.01	0.1	1	10	100
0	1.048	0.8483	0.2422	0.1489	0.1487	0.1434
0.01	0.9917	0.9042	0.2973	0.1618	0.1595	0.1511
0.1	1.008	0.9388	0.3488	0.1594	0.1532	0.1511
1	1.02	0.9923	0.6269	0.1545	0.1455	0.1434
10	0.9843	0.9586	0.7908	0.1679	0.1463	0.1433
100	0.8604	0.846	0.8576	0.1784	0.151	0.1433

S.D

0.07518	0.09151	0.08499	0.05983	0.06458	0.06027
0.1496	0.06861	0.1146	0.07818	0.07578	0.06759
0.08411	0.05292	0.06217	0.06211	0.06752	0.06432
0.08811	0.1096	0.07531	0.04754	0.05854	0.06113
0.06959	0.09139	0.06064	0.05906	0.05994	0.05869
0.07765	0.06664	0.0911	0.05745	0.06433	0.05264

orf19.3874Δ

	0	0.01	0.1	1	10	100
0	0.9863	1.025	1.022	1.026	0.2287	0.1508
0.01	1.027	0.9884	1.048	1.034	0.2632	0.1599
0.1	1	1.009	1.033	1.021	0.2322	0.1592
1	0.9723	1.004	1.003	1.005	0.2602	0.1564
10	0.4751	0.5254	0.4625	0.4911	0.2153	0.1586
100	0.1739	0.1749	0.1822	0.1756	0.1775	0.163

S.D

0.03929	0.06199	0.03591	0.04858	0.03291	0.03504
0.0247	0.04368	0.01267	0.07874	0.02473	0.04052
0.01954	0.0215	0.00863	0.03186	0.02501	0.03848
0.02473	0.009663	0.02538	0.04223	0.02009	0.03758
0.017	0.01751	0.05358	0.07631	0.01905	0.03813
0.02951	0.03147	0.0354	0.03409	0.04128	0.03775

Cont.

Zinc concentration (mM)

Manganese concentration (mM)

orf19.3769Δ

Mn (mM)	0	0.01	0.1	1	10	100	S.D					
0	0.9722	1.014	1.015	0.9893	0.1756	0.1339	0.02768	0.01072	0.01468	0.02707	0.02406	0.07916
0.01	1.023	1.09	1.076	1.05	0.2668	0.138	0.1018	0.1115	0.06696	0.1138	0.1092	0.08174
0.1	1.02	1.129	1.135	1.1	0.3163	0.1513	0.09357	0.1025	0.04543	0.05549	0.1216	0.1051
1	1.033	1.113	1.107	1.158	0.3365	0.1423	0.1086	0.05611	0.06935	0.02049	0.1055	0.09253
10	1.006	1.059	1.092	1.099	0.3468	0.1074	0.1058	0.07745	0.02773	0.03425	0.1191	0.01904
100	0.8707	0.9228	0.9691	0.9641	0.254	0.1016	0.08373	0.07776	0.05621	0.0649	0.07957	0.001791

orf19.3132Δ

Mn (mM)	0	0.01	0.1	1	10	100	S.D					
0	0.9525	0.9739	0.9631	0.9555	0.2052	0.1023	0.03748	0.0189	0.04237	0.04416	0.07187	0.01182
0.01	0.9841	0.9754	0.974	0.9756	0.1958	0.1066	0.03878	0.02773	0.02682	0.006616	0.009643	0.01527
0.1	0.953	0.9624	0.954	0.991	0.1956	0.1104	0.05977	0.01649	0.06982	0.0264	0.01171	0.01334
1	0.9334	0.9713	0.9781	0.9929	0.2078	0.1059	0.04915	0.02764	0.03003	0.0398	0.0108	0.01208
10	0.9193	0.9168	0.9294	0.9621	0.1852	0.1171	0.02042	0.02066	0.02691	0.02298	0.01998	0.01485
100	0.8044	0.8358	0.8048	0.8403	0.1669	0.1111	0.05876	0.003424	0.06754	0.0382	0.007115	0.01255

orf19.52Δ

Mn (mM)	0	0.01	0.1	1	10	100	S.D					
0	1.105	1.07	1.112	1.069	0.3326	0.2417	0.0467	0.06284	0.05159	0.07308	0.05982	0.05575
0.01	1.104	1.109	1.096	1.124	0.3614	0.2289	0.03796	0.028	0.05515	0.05862	0.04834	0.01599
0.1	1.112	1.081	1.105	1.092	0.3776	0.2199	0.0921	0.0527	0.02678	0.06579	0.06682	0.02322
1	1.062	1.068	1.077	1.076	0.4115	0.2205	0.05456	0.03452	0.02559	0.05161	0.04312	0.02161
10	1.019	1.018	1.021	1.002	0.4008	0.2449	0.0533	0.04314	0.05197	0.1593	0.06464	0.04728
100	0.9198	0.9002	0.8503	0.9246	0.3791	0.213	0.06446	0.04215	0.1855	0.07723	0.07019	0.01864

Zinc concentration (mM)

Copper concentration (mM)

Wild-type

	0	0.01	0.1	1	10	100	S.D					
0	1.013	1.027	1.037	1.034	0.1618	0.1169	0.03329	0.02458	0.04088	0.02598	0.02494	0.009296
0.01	1.081	1.105	1.069	0.754	0.2448	0.1706	0.06708	0.07098	0.09663	0.1717	0.0987	0.08903
0.1	0.2588	0.2301	0.2362	0.23	0.2165	0.1615	0.1242	0.1099	0.1301	0.1201	0.1037	0.08659
1	0.1906	0.2127	0.2175	0.2325	0.2232	0.1732	0.1043	0.1053	0.1114	0.1242	0.1157	0.1098
10	0.1862	0.2258	0.2289	0.2447	0.2197	0.1686	0.08311	0.1148	0.1195	0.1363	0.0962	0.08262
100	0.2306	0.3059	0.2731	0.2894	0.241	0.2282	0.1099	0.1401	0.1068	0.1093	0.08689	0.09971

*zrc1*Δ

	0	0.01	0.1	1	10	100	S.D					
0	1.008	0.8503	0.2348	0.1038	0.09928	0.0917	0.01776	0.02804	0.08196	0.01215	0.0105	0.00303
0.01	1.033	0.975	0.3186	0.1341	0.1362	0.0933	0.09865	0.09448	0.1166	0.06108	0.08415	0.00211
0.1	0.1349	0.1647	0.1508	0.1277	0.1289	0.0985	0.06825	0.0795	0.06692	0.06954	0.07073	0.01071
1	0.1286	0.1666	0.1805	0.1503	0.1387	0.1046	0.07137	0.08771	0.1004	0.07265	0.08919	0.02228
10	0.1367	0.149	0.1488	0.1302	0.1253	0.09608	0.07911	0.08134	0.05911	0.06375	0.05626	0.00015
100	0.1415	0.1429	0.1425	0.15	0.2577	0.1421	0.002893	0.00352	0.0002828	0.01112	0.2294	0.002113

orf19.3874Δ

	0	0.01	0.1	1	10	100	S.D					
0	1.059	1.074	1.012	1.05	0.2859	0.1448	0.1591	0.1704	0.0935	0.1351	0.1807	0.06339
0.01	1.021	0.9701	0.9507	0.7958	0.2135	0.1522	0.05391	0.1001	0.07488	0.1729	0.09477	0.07211
0.1	0.1406	0.149	0.1657	0.1562	0.1628	0.1551	0.05751	0.07905	0.05189	0.06484	0.07816	0.06129
1	0.155	0.1666	0.1476	0.1536	0.1576	0.1493	0.06837	0.07593	0.05887	0.06806	0.07064	0.0661
10	0.1522	0.1602	0.1561	0.1528	0.1567	0.1614	0.06167	0.07103	0.06279	0.05779	0.06629	0.07247
100	0.2539	0.2497	0.2147	0.2597	0.1993	0.2128	0.1473	0.1262	0.07687	0.1384	0.0641	0.08261

Cont.

Zinc concentration (mM)

Copper concentration (mM)

orf19.3769Δ

	0	0.01	0.1	1	10	100	S.D					
0	0.9696	1.008	1.01	0.9703	0.1661	0.09653	0.04419	0.0333	0.04774	0.0489	0.04655	0.003505
0.01	1.006	1.021	0.9086	0.6052	0.1844	0.1045	0.05788	0.0705	0.02739	0.08243	0.1109	0.009047
0.1	0.1815	0.2205	0.2121	0.1946	0.1503	0.1195	0.1003	0.1248	0.1265	0.1145	0.1074	0.04753
1	0.1695	0.2105	0.1975	0.2051	0.1492	0.1002	0.1106	0.1238	0.1162	0.1222	0.09789	0.013
10	0.1617	0.2054	0.1934	0.1855	0.1521	0.1387	0.09479	0.1138	0.108	0.0979	0.1001	0.07777
100	0.2836	0.1998	0.2264	0.184	0.1516	0.1532	0.183	0.07324	0.09208	0.07216	0.006646	0.008086

orf19.3132Δ

	0	0.01	0.1	1	10	100	S.D					
0	0.9692	0.9421	0.9543	0.9857	0.1721	0.09478	0.05333	0.1027	0.03651	0.03448	0.0453	0.004072
0.01	0.9352	0.8909	0.8991	0.6406	0.1255	0.09773	0.07227	0.1273	0.02408	0.04817	0.008344	0.007647
0.1	0.1157	0.1151	0.111	0.1092	0.102	0.09413	0.007277	0.007801	0.007414	0.01047	0.008223	0.00685
1	0.1057	0.1037	0.1035	0.1024	0.1003	0.09483	0.01028	0.006569	0.008329	0.009532	0.009257	0.007202
10	0.102	0.1059	0.1058	0.1082	0.104	0.09903	0.007478	0.009307	0.008608	0.009441	0.008708	0.005408
100	0.1466	0.1524	0.1493	0.1639	0.1482	0.1463	0.008665	0.007936	0.00594	0.01806	0.006874	0.006388

orf19.52Δ

	0	0.01	0.1	1	10	100	S.D					
0	1.064	1.116	1.121	1.119	0.2946	0.2171	0.0643	0.03132	0.03242	0.04559	0.06169	0.04265
0.01	1.028	1.053	0.9314	0.3694	0.2418	0.2178	0.04243	0.04426	0.04301	0.04775	0.01568	0.02967
0.1	0.2042	0.2154	0.2014	0.2103	0.2091	0.2104	0.02514	0.03004	0.02371	0.0161	0.01986	0.0261
1	0.2018	0.2062	0.1966	0.2126	0.2134	0.2148	0.0288	0.02585	0.02614	0.02548	0.02968	0.02695
10	0.2025	0.2063	0.2151	0.2188	0.2142	0.21	0.02541	0.02714	0.0347	0.02533	0.02733	0.02028
100	0.2551	0.2507	0.2505	0.2553	0.2469	0.2547	0.02637	0.02996	0.0315	0.02072	0.01944	0.02918

iron concentration (mM)	Zinc concentration (mM)											
	**Wild-type**											
	0	0.01	0.1	1	10	100	S.D					
0	0.9362	0.9519	0.9378	0.9313	0.1717	0.09655	0.01806	0.02081	0.0114	0.02266	0.02006	0.00243
0.01	0.9221	0.9811	0.9856	0.9488	0.1663	0.097	0.03993	0.02386	0.03617	0.02727	0.05407	0.006373
0.1	0.8747	0.9429	0.9307	0.9659	0.2277	0.1447	0.09798	0.07033	0.1016	0.1277	0.1075	0.05884
1	0.7422	0.8107	0.6618	0.8511	0.2965	0.2314	0.197	0.09041	0.0929	0.1181	0.094	0.08278
100	0.1103	0.1118	0.1149	0.109	0.1084	0.1114	0.004664	0.003761	0.006289	0.002982	0.004117	0.003634
	*zrc1*Δ											
	0	0.01	0.1	1	10	100	S.D					
0	0.9451	0.8249	0.2471	0.1023	0.09098	0.09135	0.02097	0.05579	0.04713	0.006878	0.002799	0.002901
0.01	0.9252	0.8312	0.2821	0.1071	0.094	0.09235	0.01708	0.03721	0.0284	0.005805	0.001309	0.002373
0.1	0.8021	0.8568	0.5418	0.169	0.1189	0.105	0.03176	0.05983	0.1123	0.07488	0.03502	0.009892
1	0.7772	0.7858	0.6174	0.24	0.2417	0.1827	0.09619	0.07159	0.07552	0.06887	0.09292	0.03016
100	0.109	0.1069	0.1079	0.109	0.1106	0.1121	0.002503	0.001348	0.001269	0.001455	0.004152	0.002187
	orf19.3874Δ											
	0	0.01	0.1	1	10	100	S.D					
0	0.9113	0.9194	0.9406	0.9103	0.2239	0.1028	0.01157	0.02319	0.03142	0.03248	0.04732	0.01006
0.01	0.8851	0.8892	0.8881	0.8739	0.2053	0.1064	0.03105	0.03841	0.02761	0.05614	0.04618	0.01135
0.1	0.7852	0.765	0.8183	0.8438	0.1912	0.1199	0.03992	0.02809	0.02338	0.02693	0.04398	0.02122
1	0.5506	0.6192	0.5319	0.6787	0.2604	0.199	0.0719	0.0604	0.09056	0.02205	0.04786	0.01514
100	0.1258	0.1253	0.1297	0.1309	0.1261	0.1228	0.01911	0.006891	0.00789	0.007691	0.008335	0.01268

Cont.

Zinc concentration (mM)

Iron concentration (mM)

orf19.3769Δ

Iron \ Zinc	0	0.01	0.1	1	10	100	S.D					
0	0.9511	0.9554	0.9639	0.9444	0.2234	0.09795	0.03522	0.04938	0.04768	0.0645	0.04662	0.00894
0.01	0.9413	0.953	0.9608	0.924	0.1837	0.1026	0.04986	0.03927	0.03285	0.03423	0.01002	0.01725
0.1	0.8339	0.8527	0.8719	0.9114	0.193	0.111	0.03837	0.01119	0.0118	0.04039	0.01755	0.01602
1	0.6237	0.6949	0.6172	0.8053	0.2786	0.1859	0.1003	0.009269	0.09135	0.0221	0.03975	0.01819
100	0.1234	0.133	0.1327	0.136	0.1273	0.1226	0.01875	0.02143	0.01928	0.02045	0.01645	0.01854

orf19.3132Δ

Iron \ Zinc	0	0.01	0.1	1	10	100	S.D					
0	0.9418	0.8876	1.028	0.9514	0.2374	0.1178	0.02519	0.1189	0.1525	0.0404	0.0843	0.02148
0.01	0.9545	0.9391	0.9565	0.945	0.2094	0.1309	0.02233	0.0151	0.03878	0.0353	0.009045	0.03332
0.1	0.8561	0.8607	0.8781	0.8598	0.2332	0.1418	0.01415	0.02952	0.01116	0.04777	0.04518	0.0329
1	0.6285	0.6725	0.6014	0.7458	0.3139	0.213	0.02614	0.04067	0.01965	0.07369	0.0494	0.03976
100	0.1746	0.1748	0.1836	0.1939	0.1953	0.1671	0.03424	0.01687	0.007687	0.02181	0.02246	0.04922

orf19.52Δ

Iron \ Zinc	0	0.01	0.1	1	10	100	S.D					
0	1.043	1.008	1.001	0.996	0.3135	0.1596	0.06436	0.09258	0.1167	0.08266	0.0791	0.1116
0.01	0.9607	0.989	0.9378	0.9798	0.2474	0.1365	0.05506	0.09591	0.03598	0.06924	0.0287	0.04084
0.1	0.8593	0.8578	0.8823	0.9038	0.279	0.1474	0.04794	0.07683	0.07279	0.06107	0.03553	0.01762
1	0.6903	0.722	0.6376	0.826	0.3728	0.2344	0.03864	0.1499	0.07675	0.1312	0.0574	0.03122
100	0.1245	0.1256	0.1552	0.154	0.151	0.1382	0.02492	0.02123	0.04418	0.02537	0.01924	0.02227

Figure S5. Effect of zinc and manganese, copper or iron on the growth of wild-type *C. albicans* and ZnT deletion mutants. Optical densities of overnight cultures were adjusted to 0.05 than incubated for 24 hrs in S.D media containing indicated metal concentrations. The data showed no difference in the growth pattern. Data are the mean of two independent experiments, in duplicate, and standard deviation (S.D) values in the right column.
